# The DinG exonuclease acts as a primary quality controller to remove unprocessed ribosomal RNAs

**DOI:** 10.1093/nar/gkaf1446

**Published:** 2026-01-06

**Authors:** Karolis Vaitkevičius, Jörgen Johansson

**Affiliations:** Department of Molecular Biology, Umeå University, Umeå, Sweden; Laboratory for Molecular Infection Medicine Sweden, Umeå University, Umeå, Sweden; Umeå Centre of Microbial Research, Umeå University, Umeå, Sweden; Department of Molecular Biology, Umeå University, Umeå, Sweden; Laboratory for Molecular Infection Medicine Sweden, Umeå University, Umeå, Sweden; Umeå Centre of Microbial Research, Umeå University, Umeå, Sweden

## Abstract

Bacteria lacking DEAD-box RNA-helicases often show reduced growth and aberrant maturation of ribosomal subunits resulting in fewer active ribosomes. Here, we show that the slow growth observed in a strain lacking the RNA-helicase CshC in the bacterial pathogen *Listeria monocytogenes* can be suppressed by mutations in the exonuclease DinG. A strain lacking both CshC and DinG increased the number of mature and active ribosomes compared to the parental Δ*cshC* mutant. DinG acts as a 3′- to 5′-exoribonuclease, targeting immature, unprocessed ribosomal RNA *in vitro* and *in vivo* while leaving processed rRNA undisturbed. In addition, DinG directly or indirectly interferes with the ribonuclease M5 mediated pre-5S rRNA processing. We suggest that DinG acts as a primary ribosome quality control ribonuclease that initiates degradation of unprocessed rRNA.

## Graphical Abstract

**Graphical Abstract:**
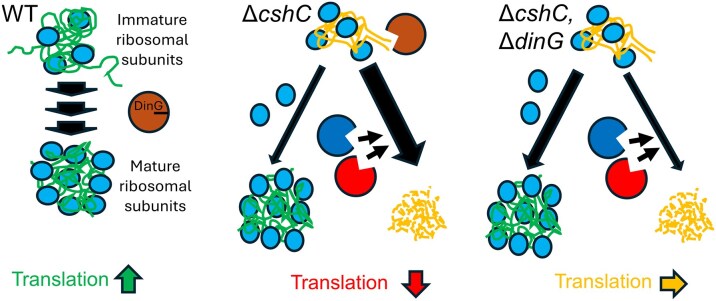

## Introduction

Ribosome maturation is a highly complex and well-orchestrated process involving many different factors [1]. The 30S and the 50S subunits of the bacterial ribosome mature independently, before forming an actively translating ribosome on the target transcript. During ribosomal subunit assembly, ribosomal RNA (rRNA) matures in a hierarchical manner. After processing of the primary transcript, the pre-rRNAs fold into secondary structures enabling binding of a first set of ribosomal proteins (r-proteins). The binding of the first set of r-proteins alters the local rRNA folding allowing sequential binding of other r-proteins. In *Bacillus subtilis*, r-proteins assist in not only maturation but also different assembly factors [1, 2]. For instance, efficient assembly of the 50S ribosomal subunit in *B. subtilis* involves sequential as well as parallel processes that converge in an assembly intermediate. To form mature 50S subunits, different assembly factors are needed, such as RbgA, YphC, and YsxC [3–6]. The depletion of any of these factors “locks” the ribosome into an intermediate assembly state and the bacterium will grow slower or die due to inefficient or inhibited translation. Surprisingly, although requiring extended incubation times, higher salt concentrations and elevated temperatures, the ribosomal subunits can mature *in vitro*, also in the absence of assembly factors [7, 8]. Once r-protein assembly is completed, the removal of 5′ and 3′ extensions from each of the three rRNA species 16S, 23S, and 5S rRNA acts as a quality control step to guarantee the ribosomal subunits are functional and stable. For example, YqfG, required for final processing of the 16S rRNA 3′-precursor, is essential in *B. subtilis*, unless also another ribonuclease, RNase R, is deleted [9]. RNaseR has been suggested to act as a quality control ribonuclease and remove defective ribosomal subunits [9, 10].

DEAD-box RNA-helicases (RNA-helicases) are conserved RNA binding proteins that are important to unwind perturbing structures in the RNA molecule [11]. Through a conserved core composed of two domains with a RecA-fold, bacterial RNA-helicases display an ATP-dependent RNA binding and RNA-dependent ATP-hydrolysis. Several studies have shown that RNA-helicases are involved in bacterial ribosome maturation and rRNA processing. The Gram-positive bacterial pathogen *Listeria monocytogenes* encodes four different RNA-helicases, CshA (Lmo0866), CshB (Lmo1450), CshC (Lmo1722), and CshD (Lmo1246) that show overlapping, but also distinct activities [12]. Bacteria lacking one or several of the RNA-helicases show growth defects (particularly at low temperatures), reduced motility, a defective ability to respond to different stresses and a reduced virulence gene expression and ability to infect eukaryotic cells [12–16]. Until now, the fate of immature and unprocessed ribosomal subunits in bacteria lacking certain RNA-helicases has not been fully addressed: are they allowed to eventually mature into functional ribosomal subunits or are they removed through specific pathways?

In this work, we identified suppressor mutations in the gene encoding the 3′ to 5′ exoribonuclease DinG that improved growth of the Δ*cshC* mutant. In contrast to the parental Δ*cshC* mutant, the Δ*cshC*, Δ*dinG* double mutant had an increased number of mature 70S ribosomes and polysomes and also showed an improved translation efficacy. *In vivo* and *in vitro* experiments showed that DinG rapidly initiated degradation of unprocessed rRNA, while maintaining processed and mature RNA intact. Based on our results, we suggest that DinG acts as a primary quality control nuclease to remove ribosomal subunits carrying incompletely processed rRNA.

## Materials and methods

### Strains and plasmid construction

*Escherichia coli* and *L. monocytogenes* strains are listed in Supplementary Table S1. *Escherichia coli* was grown in LB and *L. monocytogenes* in brain heart infusion (BHI) at indicated temperatures, unless otherwise noted. Where needed, antibiotics were included in the growth media at these final concentrations: carbenicillin 100 µg/ml, kanamycin 50 µg/ml, nalidixic acid 50 µg/ml, colistin sulfate 10 µg/ml. Cloning was performed using standard techniques [17] using primers listed in Supplementary Table S2. Plasmids used in this study are listed in Supplementary Table S3. For expression of *dinG* in *Listeria*, the gene was cloned into an isopropyl β-D-1-thiogalactopyranoside (IPTG) inducible pIMK3 vector [18]. The resulting constructs were transferred to *L. monocytogenes* by conjugation with *E. coli* S17-1 strain carrying these plasmids [19]. Transconjugants were selected by plating on BHI plates containing kanamycin, colistin sulfate, and nalidixic acid. For protein expression and purification *dinG* was cloned with a C-terminal hexahistidine tag into pET11a vector and transferred into *E. coli* BL21(DE3)pLysS (Novagen) by electroporation.

### Isolation of suppressor mutants and whole genome sequencing

The *L. monocytogenes* mutant Δ*cshC* growth is cold sensitive [16]. Occasionally larger colonies appear when this strain is cultivated on BHI-agar plates at low temperatures. Five such colonies that appeared from independent cultures grown at 16°C were picked. The clones were purified by re-streaking on BHI-agar plates at least three times and growing at 37°C. Chromosomal DNA from each of these derivatives was sequenced by MicrobesNG (https://microbesng.com).

### Generation time estimation

Growth rate comparison for *L. monocytogenes* derivatives used in this work was done in BHI liquid medium at 20°C. Pre-cultures grown in BHI media with shaking at 37°C were used as inoculum. The bacteria were grown in 96-well flat-bottomed microtiter plates with 200 µl media volume per well. Incubation was performed in Tecan Spark multimode microplate reader equipped with a Te-Cool cooling module and a humidity cassette. Shaking conditions were 3 mm amplitude orbital shaking at 180 rpm. The light absorbance at 600-nm wavelength was measured every 15 min. The growth curves were plotted in Excel and the data intervals that correspond to exponential growth were fitted with exponential growth equation in GraphPad Prism v10 to calculate the generation times. Three biological replicates were used.

For WT, Δ*dinG*, Δ*cshC*, and Δ*dinG* Δ*cshC* strains generation times were also estimated using the colony forming units counting. Bacteria were grown in 25 ml BHI medium with 160 rpm shaking at 20°C. Samples were collected at four different time-points in the exponential growth interval that lies between culture density values of 0.1–1 of A_600_ measured on Amersham Ultrospec 2100pro spectrophotometer (Cytiva). Culture samples were diluted 10^5^ or 10^6^ fold in phosphate-buffered saline and 100 µl volume of diluted bacteria was plated on LA plates. Two plates were used for each dilution and the CFU data was collected from four biological replicates of each tested strain. Generation times were calculated by fitting the exponential growth equation to the bacterial concentration plotted over time using GraphPad Prism v10 software.

### RNA isolation

To isolate total RNA from *Listeria*, liquid cultures were grown to a defined growth phase, typically optical density of 0.5 absorbance units at 600 nm wavelength, mixed with 0.2 volumes of 5% phenol in 95% ethanol [20], and bacteria harvested by centrifugation. Bacterial pellets were frozen in liquid nitrogen and stored at −80°C. RNA from *L. monocytogenes* was isolated using a modification of guanidinium thiocyanate–phenol–chloroform extraction [21].

RNA extraction from sucrose gradient fractions or enzymatic reaction mixtures was performed using Directzol RNA Miniprep Kits (Zymo Research). If necessary, DNase treatment and cleanup was done using RNA Clean & Concentrator kits (Zymo Research). To extract RNA from agarose gels the Zymoclean Gel RNA Recovery Kit (Zymo Research) was used.

### Nucleic acid electrophoresis

The separation of rRNA was performed in mixed Synergel (Carl Roth) and agarose (0.9% and 0.7%, respectively) gels in 1× TBE [45 mM Tris-borate, pH 8, 1 mM ethylenediaminetetraacetic acid (EDTA)] and the rRNA was visualized using ethidium bromide as previously described [22].

### Northern blotting

For northern blotting, equal amounts of 3–20 μg of total RNA was separated in a formaldehyde agarose gel prior to blotting as described [21, 23]. The Hybond-N membrane (GE Healthcare) was subsequently hybridized with oligonucleotide probes end-labeled with ^32^P γ-ATP (Revvity or Hartmann Analytic) using polynucleotide kinase (Thermo Fisher Scientific). Labeled DNA oligonucleotide probes were cleaned using ProbeQuant G-50 Micro Columns (Cytiva) prior to hybridisation. Northern blots were developed, and band intensities were measured in the Typhoon FLA9500 scanner (GE). Oligonucleotide DNA probes for detection of specific RNAs are listed in Supplementary Table S2.

### Precursor rRNA stability assay

*Listeria* cultures were grown in BHI at 20°C until A_600 _= 0.5. Rifampicin was added to a final concentration of 1 mg/ml from a 200 mg/ml stock solution in dimethyl sulfoxide (DMSO). At time points between 0 and 24 h, culture samples of 10 ml volume were removed and mixed with 2 ml STOP solution (5% phenol in ethanol). Bacteria were pelleted by centrifugation for 10 min at 7000 × *g* and pellets were stored at −80°C until RNA isolation. Four micrograms of total RNA were separated on 0.7% agarose/0.9% Synergel in 1× TBE. After electrophoresis, gels were stained in 1 µg/ml ethidium bromide, and imaging was made in ChemiDoc XRS (BioRad). The intensities of RNA bands were determined using ROI manager tool from ImageJ2 [24]. The precursor rRNA stability experimental data were fitted with exponential decay curve using GraphPad Prism v10 to estimate the half-lives of these RNA species.

### Ribosome profiling by sucrose gradient centrifugation

Ribosome and polysome profiles using sucrose gradient centrifugation were done essentially as described previously [25–27]. No DNase or other nuclease was added to the lysates. *L. monocytogenes* strains were grown in 300 ml BHI broth at 20°C with 160 rpm shaking. Overnight cultures grown at 37°C were used as inoculum by diluting to A_600 _= 0.02 into fresh medium equilibrated to 20°C. When cell density reached an A_600 _= 0.5, 100 µg/ml chloramphenicol was added to stop translation, cultures were cooled down on ice and harvested by centrifugation at 8000 × *g* for 15 min. The bacterial pellets were frozen in liquid nitrogen for storage if necessary. The pellets were suspended in 0.75 ml solution R (10 mM Tris–Cl, pH 7.5, 60 mM KCl, 10 mM MgCl_2_) with 6 mM 2-mercaptoethanol, 100 µg/ml chloramphenicol and 1× Complete protease inhibitor mix, EDTA-free (Roche). Bacteria were lysed by FastPrep24 (MP Biomedicals) beating with 0.1 mm zirconia beads for four cycles of 20 s duration at 6 m/s speed. Triton X-100 reduced (Merck, X100RS) was added to the disrupted bacteria at final concentration of 0.1% and sodium deoxycholate was added to 0.16%. Insoluble debris was removed by centrifugation at 10 000 × *g* for 10 min. Lysates were frozen in liquid nitrogen and stored at −80°C. For polysome fractionation equal amounts of thawed lysate samples corresponding to 100 µl of 100 A_260_ units were loaded onto 10%–40% sucrose gradients prepared in solution R. After centrifugation at 35 000 rpm for 3.5 h in Beckman SW45Ti rotor, the separated ribosome profiles were read by measuring light absorbance at 260 nm using a Piston Gradient Fractionator (BioComp Instruments). In some experiments, 15%–30% sucrose gradients and centrifugation at 22 000 rpm for 19 h was used to improve ribosome precursor separation.

### Protein electrophoresis, western blotting

Protein samples were analyzed by sodium dodecyl sulfate–polyacrylamide gel electrophoresis (SDS-PAGE) [28] and/or western blotting on polyvinylidene fluoride (PVDF) membrane [29]. For extraction of proteins from sucrose gradients, the methanol/chloroform method was used [30]. Proteins were separated in 10% polyacrylamide gels and either stained with Coommassie Brilliant Blue or transferred onto a PVDF membrane using a tank transfer apparatus (Bio-Rad). Development of the membrane essentially followed the protocol of the ECL Prime Western blotting kit (GE Healthcare) using a primary 6x-His tag monoclonal antibody [(HIS.H8), MA1-21315, Thermo Fisher Scientific], and a peroxidase (HRP)-conjugated polyclonal rabbit anti-mouse secondary antibody (P0260, DAKO). Measurement of luminescence signal was carried out in Amersham Imager 680 (GE).

### Puromycin incorporation assay

Puromycin minimal inhibitory concentration (MIC) for the four strains used in the experiment was determined using a broth microdilution method as defined by European Committee on Antimicrobial Susceptibility Testing guidelines https://www.eucast.org/bacteria/methodology-and-instructions/mic-determination/. Briefly, BHI medium was inoculated with *L. monocytogenes* at 5 × 10^5^ CFU/ml into round-bottom 96-well plates to test the growth in presence of serially diluted puromycin. During the incubation at 20°C, the presence or absence of visible growth was scored for up to 96 h. At these conditions, the puromycin MIC for *L. monocytogenes* was 128 µg/ml. For puromycin incorporation assay, *Listeria* strains were grown in BHI liquid medium at 20°C and 160 rpm shaking. When bacteria reached the optical density of A_600_ = 0.5, puromycin (Merck, P7255) was added to a final concentration of 25 µg/ml from a 10 mg/ml stock solution in water. After 30 min incubation, culture samples of 10 ml volume were mixed with 2 ml STOP solution (5% phenol and 0.6 mg/ml chloramphenicol in ethanol) and cooled on ice. Bacteria were pelleted by centrifugation for 10 min at 7000 × *g* and pellets were stored at −80°C until lysate preparation for protein electrophoresis. Lysates were prepared using mutanolysin method [31]. Viscosity of the samples was reduced by 30-min sonication with 30 s ON and 30 s OFF cycles at 0°C in Bioruptor Pico (Diagencode). Insoluble debris was removed by 15-min centrifugation at 18 000 × *g*. Protein concentration was determined with Qubit Protein Assay (Thermo Fisher Scientific). Equal protein amount of 20 µg from different *Listeria* strains was loaded on NuPAGE BisTris 4%–12% gradient Mini Protein Gels (Thermo Fisher Scientific) for electrophoresis. Separated proteins were transferred onto Immobilon-P PVDF membrane (Merck-Millipore) using Novex wet transfer apparatus (Thermo Fisher Scientific). Equal protein transfer was confirmed by staining the membrane with 0.01% Ponceau in 0.5% acetic acid [32]. Incorporated puromycin was detected using anti-puromycin primary antibody (Merck, clone 12D10, MABE343) and anti-mouse IgG -peroxidase secondary antibody (Merck, A9044) using ECL detection reagent (Cytiva, RPN2105). The luminescence of blots was recorded in LAS4000 imager (Fuji) and signal intensities were measured using ImageJ2 ROI analysis tool.

### Protein purification

Hexahistidine tagged DinG and its derivatives D10A, E12A, or D155Y (exonuclease deficient) and E465A (ATPase deficient) were expressed from pET11a vector in *E. coli* BL21(DE3) pLysS strain. Bacterial cultures were started by diluting freshly grown inoculates into 500 ml LB medium containing 150 µg/ml carbenicillin and 12.5 µg/ml chloramphenicol to an optical density at 600 nm (A_600_) of 0.01. Cultures were grown shaking at 200 rpm to an A_600_ of ~0.3–0.6 at 37°C, cooled on ice before the continued growth at 25°C in presence of 0.4 mM IPTG for 9–10 h. Bacteria were harvested by centrifugation at 5000 × *g* for 30 min. The pellet was washed by keeping 1 h on ice suspended in 1/10 culture volume of solution W (50 mM Tris–Cl, pH 8, 200 mM NaCl, 5 mM EDTA, 20% sucrose with freshly added 0.1 mg/ml lysozyme, 1 mM PMSF, and 1 mM benzamidine). After centrifuging at 5000 × *g* for 15 min, the pellet was either processed further or frozen in liquid nitrogen for storage. The bacteria were lysed by suspending in solution L (50 mM Tris–Cl, pH 8, 200 mM NaCl, 30 mM imidazole) with 1 mM each of PMSF and benzamidine. The viscosity of the lysate was reduced by either several passages through 21G and 23G needles or brief sonication. The lysate was cleared by centrifugation at 50 000 × *g* for 1 h at 4°C. The supernatant was loaded onto 5 ml HisTrap FF (Cytiva) column and proteins eluted with an imidazole gradient using solution A (50 mM Tris–Cl, pH 8, 500 mM NaCl, 30 mM imidazole, 10% glycerol, 1 mM PMSF) and B (50 mM Tris–Cl, pH 8, 500 mM NaCl, 500 mM imidazole, 10% glycerol, 1 mM PMSF). The peak DinGH_6_ or its derivative protein fractions were concentrated with a Vivaspin 20 5 kDa cutoff cartridge (Cytiva) and separated on a HiPrep 16/60 Sephacryl S-200 HR column (Cytiva) equilibrated with solution S (50 mM Tris–Cl, pH 7.5, 500 mM NaCl, 10% glycerol, 1 mM PMSF). The purest protein fractions were combined, concentrated, and frozen in aliquots in liquid nitrogen.

### Nuclease activity assays

The nuclease activity of DinG was assessed in a reaction buffering solution D (20 mM Tris–Cl, pH 7.5, 50 mM NaCl, 5 mM MgCl_2_, and 0.1 mg/ml nuclease-free BSA). RNA and DNA oligonucleotide substrates were ordered from EuroFins, labeled at 5′ end with [^32^P] γ-ATP (Revvity or Hartmann Analytic) using polynucleotide kinase, and cleaned-up using Oligo Clean & Concentrator kit (Zymo Research). For 3′ end labeling the RNA was ligated to [^32^P]pCp (Hartmann Analytic) using T4 RNA ligase 1 (NEB, M0204L) and cleaned-up using Oligo Clean & Concentrator kit (Zymo Research). The RNA oligonucleotide pre23S_ends_5xPTO with 5 consecutive phosphorothioate linkages introduced at the 3′ end was ordered from EuroFins. The labeled structured RNA oligonucleotide substrates pre23S_ends, pre23S_ends_blunt and pre23S_ends_5xPTO (Supplementary Table S2) were heated to 80°C and allowed to slowly reach the reaction temperature before initiating the activity assay. The reaction mixtures containing DinG and radioactively labeled oligonucleotide substrates were at defined time points mixed with a Gel Loading Buffer II (AM8547, Thermo Fisher Scientific) and frozen in liquid nitrogen for temporary storage. The samples were run in denaturing 8 M urea, 20% 19:1 acrylamide/bis-acrylamide gels in 1 × TBE. Autoradiography on storage phosphor screens was read using a Typhoon FLA9500 scanner (GE).

The activity toward ribosomes and their precursors was estimated by directly adding purified DinG to a total lysate of *L. monocytogenes* Δ*cshC*Δ*dinG* which harbors high levels of rRNA precursors and lacks endogenous DinG activity. RNA from these reaction mixtures was extracted using Directzol RNA Miniprep Kits (Zymo Research). The RNase activity of DinG was assessed in Synergel modified agarose gels and 1 µg/ml ethidium bromide staining.

DinG activity in precursor rRNA enriched fraction from sucrose gradient separated lysate was performed similarly, but solution exchange was done using Vivaspin 500, 100 kDa MWCO Polyethersulfone spin columns (28–9322-37, Cytiva) to remove sucrose and other small molecules. After electrophoresis RNA was detected in gels using SYBR Gold (S11494, Thermo Fisher Scientific) staining due to lower amount of initial material.

### Direct RNA sequencing

The sequencing of gel-purified RNA fragments was performed on a Minion Mk1B device controlled by MinKNOW 23.07.15 or later software. Oxford Nanopore Flongle Flow Cells (R9.4.1) were used according to modified manufacturer’s instructions (https://www.protocols.io/view/flongle-directrna-library-preparation-kqdg35847v25/v1). The reads were mapped to the *L. monocytogenes* strain EGDe genome with minimap2 -ax splice -un -k14 [33]. The sorted and indexed reads were visualized using Integrative Genomics Viewer (IGV) [34]. Sequencing data files are deposited in NCBI under a BioProject PRJNA1256696.

### Protein structure prediction

The protein structure of *L. monocytogenes* DinG was predicted using AlphaFold 3 [35] (https://alphafoldserver.com/) using these putative cofactors/substrates: 3 Mg2 + ions, ATP and pre23S_ends RNA oligonucleotide (Supplementary Table S3). The predicted structure was visualized using UCSF Chimera 1.18 or ChimeraX 1.9 software [36, 37].

## Results

### Compensatory mutations in the gene encoding the DinG nuclease suppress the slow growth rate observed in bacteria lacking the DEAD-box RNA-helicase CshC.

**Figure 1.**
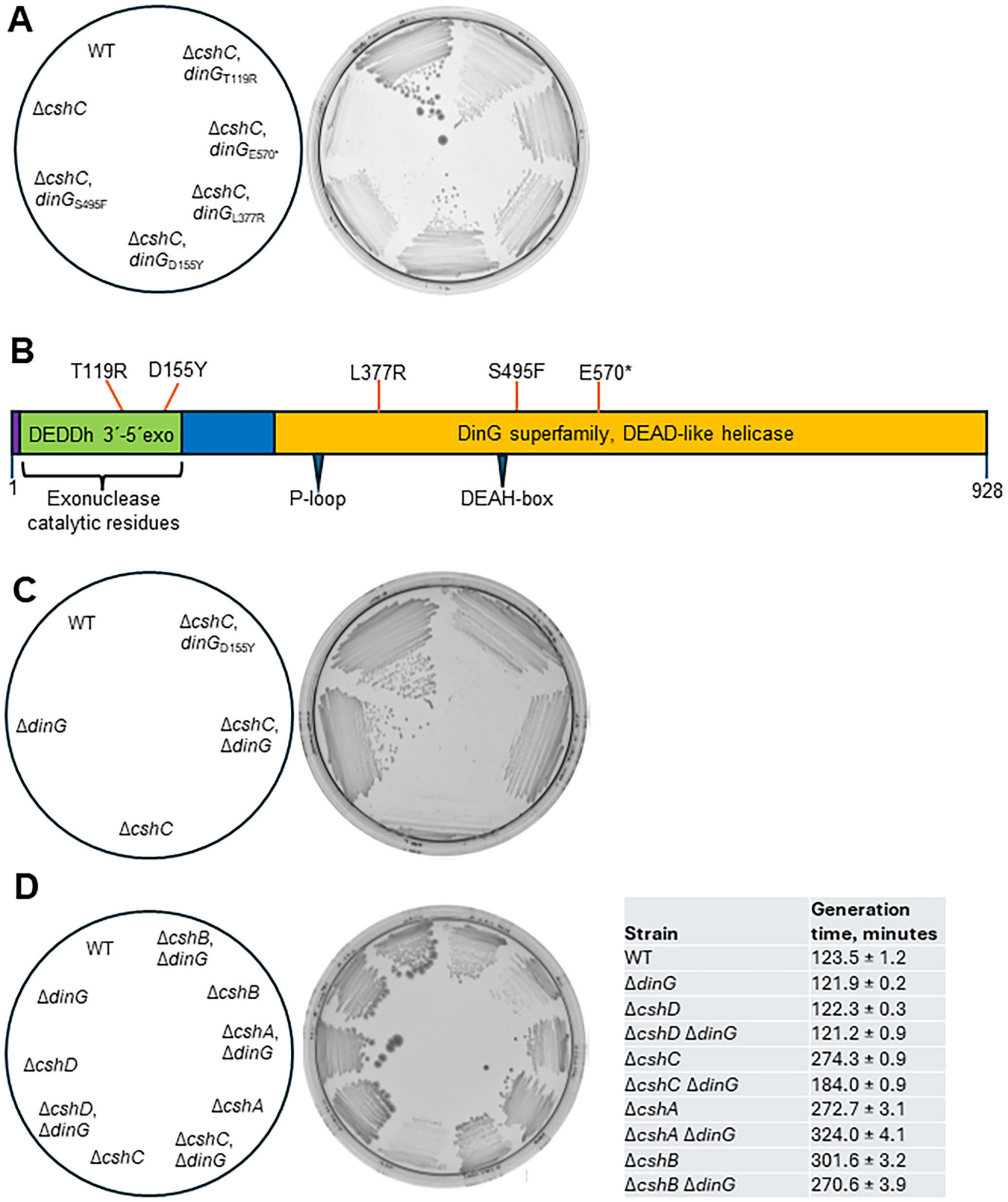
Mutations in the gene encoding the DinG nuclease suppress the cold sensitivity phenotype observed in bacteria lacking RNA-helicases. (**A**) Growth of Δ*cshC* suppressor mutants. Isolated suppressor mutants were plated on BHI agar plates and incubated at 16°C for two weeks. (**B**) Schematic illustration of DinG. The exonuclease domain is shown in green and the helicase domain (including a DEAH and a P-loop site) in orange. Letters and numbers above the protein denote site of amino acid substitutions in suppressor mutants. An asterisk denotes a stop codon at that position of DinG. See also Supplementary Fig. S1. (**C**) Growth of the Δ*dinG* mutant and derivatives. Isolated suppressor mutants were plated on BHI agar plates and incubated at 16°C for two weeks. (**D**) Growth of the Δ*dinG* deletion in combination with deletions of genes encoding other DEAD-box RNA helicases. Isolated suppressor mutants were plated on BHI agar plates and incubated at 16°C for two weeks. Inset shows generation time of indicated strains in liquid BHI-cultures at 20°C over time. ± denotes standard deviation (SD) (*n* = 3).

We have previously characterized the function of the four DEAD-box RNA-helicases in *L. monocytogenes*. Absence of three of these four RNA-helicases reduces growth rate of the bacterium, particularly at low temperatures [12]. A previous study showed that the RNA-helicase CshC (Lmo1722) interacts with the 50S subunit of the ribosome through its N-terminus [16]. A strain lacking CshC was viable but showed a disturbed ribosomal maturation and a dramatically reduced growth rate at 16°C [12, 16]. To get further insights into the function of CshC, we isolated suppressor mutants in the Δ*cshC* mutant showing improved growth on agar-plates at low temperatures. Five suppressor mutants were isolated, which all grew better compared to the parental Δ*cshC* mutant at 16°C (Fig. 1A). Whole genome sequencing revealed that all isolated strains harbored mutations in the gene *dinG* (*lmo1899*), leading to diverse amino acid substitutions or a frame shift producing a truncated protein (Fig. 1B and Supplementary Table S4). Lmo1899 (UniProt entry Q8Y604 [38]) shows a high level of identity to DinG in *Staphylococcus aureus* [39]. DinG in *E. coli* has been suggested to unwind stalled replication forks by showing a DNA/RNA helicase activity and CasDinG is essential for type IV CRISPR immunity [40, 41]. As a difference to *E. coli* DinG, DinG in *S. aureus* and other firmicutes lacks a FeS cluster domain and is therefore unable to unwind DNA [39, 42, 43], although recent work demonstrated that SaDinG can unwind DNA duplexes [44]. DinG in *S. aureus* (as DinG in *L. monocytogenes*) has acquired an extra N-terminal nuclease domain showing 3′- to 5′-exonuclease activity (Fig. 1B and Supplementary Fig. S1) [39]. The nuclease part of the DinG protein belongs to a superfamily of DnaQ-like or DEDD exonucleases that include enzymes involved in proofreading during DNA replication, DNA repair, ribosomal and transfer RNA processing, and others [45]. The exonuclease active site of these nucleases is four invariant acidic residues DEDD important in catalysis and the exonuclease active site of DinG is D_10_E_12_D_96_D_155_ (Fig. 1 and Supplementary Fig. S1). Interestingly, *dinG*_D155Y_ which is one of the *dinG* mutations that partially suppresses the cold sensitive phenotype of the *L. monocytogenes* Δ*cshC* mutant, changes the fourth residue of the exonuclease signature motif (Fig. 1A and B). We therefore reasoned that inactivation of the putative nuclease activity of DinG may be the reason behind the observed phenotype. In line with this hypothesis, Alpha-fold structural prediction of the DinG protein suggested that all amino acid substitutions identified through the suppressor mutant screening were located at the site of the protein facing the RNA molecule and/or close to the catalytic site (Supplementary Fig. S1). This prediction was strengthened by the recent determination of the crystal structure of DinG protein in *S. aureus*, where part of the ssDNA used in that study overlapped with the single-stranded part of the RNA substrate that we used in the Alpha-fold prediction, at the exonuclease domain of DinG [44]. In that study, a part of the ssDNA that interacted with the exonuclease domain of DinG, overlapped with the single-stranded part of the RNA substrate (3′-part of the pre-23S rRNA) that we used in the alpha-fold prediction [44].

To verify that the growth restoration was caused by an inactivated DinG, a Δ*cshC*, Δ*dinG* double deletion mutant was created. The double mutant showed improved growth compared to the Δ*cshC* mutant as scored on plate, and the generation time was shortened in liquid cultures (Fig. 1C and Supplementary Fig. S2). Our results thus suggest that the slow growth phenotype observed in a Δ*cshC* mutant can be suppressed by deletion of the *dinG* gene. Despite testing various growth conditions, we have been unable to identify a phenotype associated with the Δ*dinG* single knock-out mutant.

### The deletion of *dinG* suppresses the growth of *L. monocytogenes* lacking CshB

As for the Δ*cshC* mutant, strains lacking the RNA-helicases CshA and CshB (but not CshD) showed a growth deficiency and formed defective ribosomal subunits in *L. monocytogenes*, particularly at low temperatures [12, 16]. However, the ribosomal maturation patterns contrasted in the different RNA-helicase mutants indicating that the RNA-helicases affected distinct phases of ribosomal maturation either directly or indirectly [12]. We therefore asked whether the improved growth observed by deleting *dinG* in the Δ*cshC* mutant also could be observed in strains lacking other RNA-helicases. To examine this, Δ*cshA*, Δ*dinG*; Δ*cshB*, Δ*dinG*; and Δ*cshD*, Δ*dinG* double mutants were created and their growth on agar-plates examined as well as their generation time in liquid culture. In comparison with the Δ*cshB* single deletion mutant, the Δ*cshB*, Δ*dinG* double mutant showed a slightly improved growth but not as pronounced as the Δ*cshC*, Δ*dinG* double mutant (Fig. 1D). In contrast, deletion of DinG in the Δ*cshA* mutant increased the generation time. Since CshB and CshC affect different steps in ribosomal maturation [12], our results suggest that the effect of the DinG deletion depends on which RNA-helicase is missing and could couple DinG-activity to a particular stage of ribosomal maturation.

### The absence of DinG in the Δ*cshC* mutant restores the level of active 70S ribosomes

**Figure 2.**
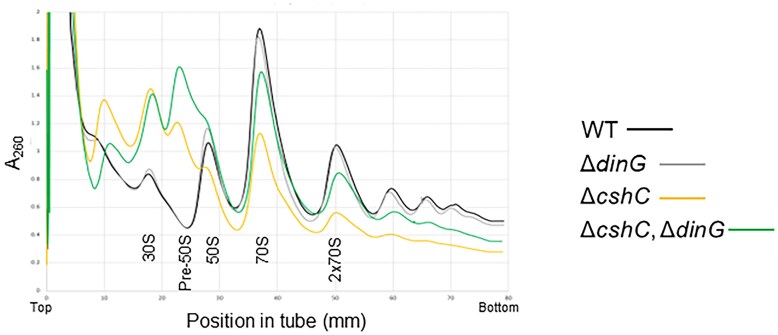
Deleting *dinG* in a *cshC* mutant increases the number of polysomes. Polysome profiles of EGDe (WT); Δ*dinG*; Δ*cshC* and Δ*cshC*, Δ*dinG* mutants. Bacterial cultures of indicated strains were grown at 20°C, before being lysed and separation of supernatant by ultracentrifugation in 10%–40% sucrose gradients. Light absorbance at 260 nm wavelength was measured to visualize ribosome species and polysome patterns in sucrose gradients. Identity of peaks are shown (30S, pre-50S, 50S, 70S, and polysomes, respectively). The graph is a representative of three sets of replicates. See also Supplementary Fig. S3.

To examine whether the improved growth observed in the Δ*cshC*, Δ*dinG* double mutant correlated with an increased number of mature and active ribosomes, ribosome maturation was examined by sucrose gradient profiling. As observed previously, absence of CshC dramatically decreased the number of mature 70S ribosomes and polysomes but increased the amount of 30S subunits and pre-50S subunits (Fig. 2) [12, 16]. The amount of 30S subunits and pre-50S ribosome subunits was high also in the Δ*cshC*, Δ*dinG* double mutant, but strikingly, the number of actively translating 70S ribosomes (monosomes and polysomes) was increased, reaching levels between those of WT and Δ*cshC* mutant (Fig. 2). To further study ribosome maturation, rRNA processing in the Δ*cshC* mutant and the Δ*cshC*, Δ*dinG* double mutant was investigated. Samples from the different sucrose gradient fractions were separated on agarose gels (Supplementary Fig. S3). In both mutants, the fractions possessing the 30S and 50S subunits in the Δ*cshC*, Δ*dinG* double mutant all showed a high degree of unprocessed 16S and 23S rRNA. In contrast, the active 70S ribosome essentially carried processed rRNA (Supplementary Fig. S3).

### The translation efficiency is improved in the Δ*cshC*, Δ*dinG* double mutant as compared with the Δ*cshC* single mutant

**Figure 3.**
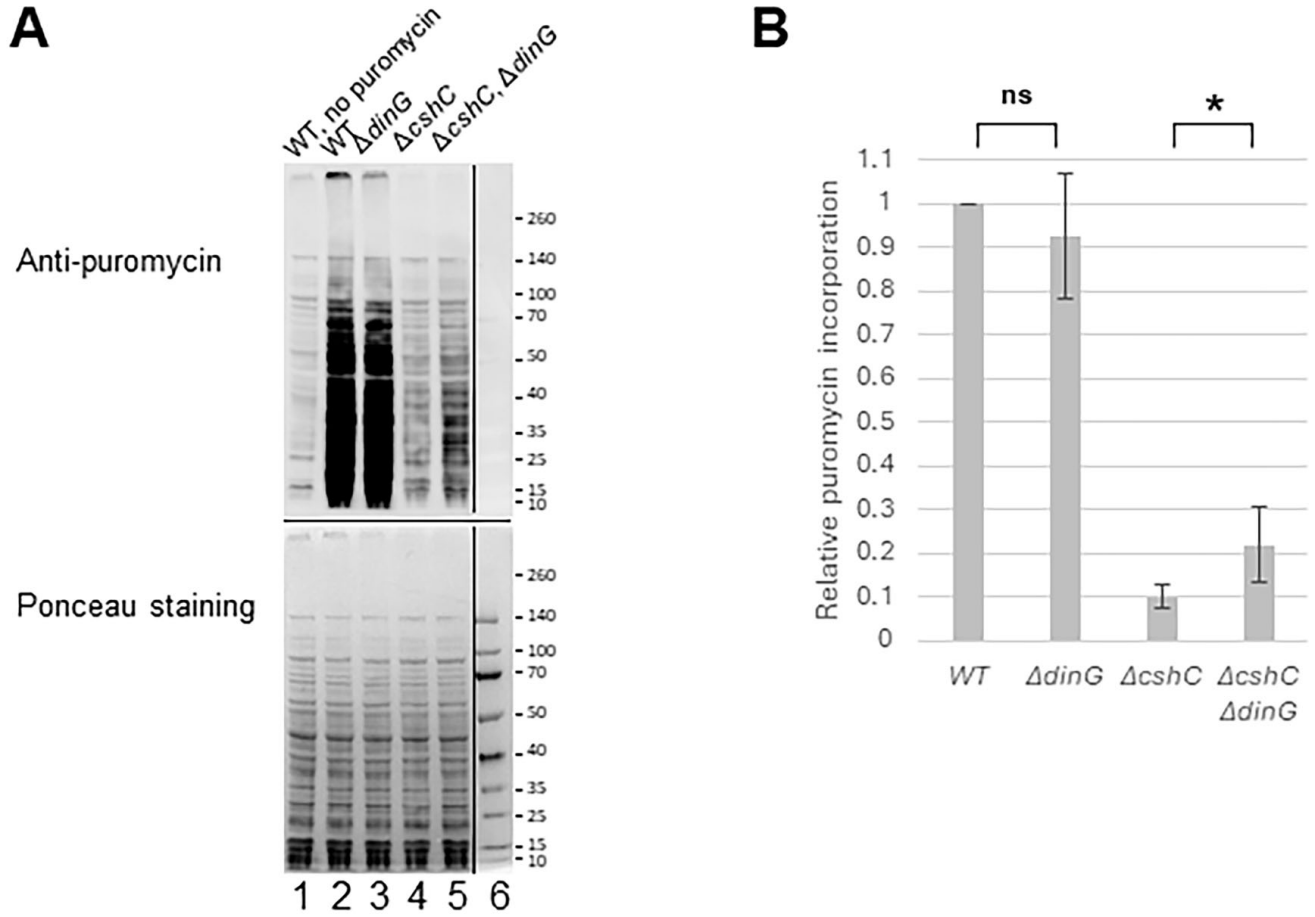
Overall translation is reduced in the strain lacking CshC. (**A**) Indicated strains were grown at 20°C until A_600 _= 0.5, when puromycin (25 µg/ml) was added. After 30 min, the cultures were harvested, proteins isolated, and separated by SDS–PAGE, before western blotting using an anti-puromycin antibody to monitor translation efficiency. Puromycin is incorporated into elongating peptide chains. (**B**) Quantification of puromycin incorporation from panel (A). Puromycin incorporation is relative to WT (*n* = 6). The error bars indicate standard deviation, Unpaired two-tailed *t-*test, *P < .05.

Since the Δ*cshC*, Δ*dinG* double mutant grew faster and contained an increased number of mature 70S ribosomes and polysomes as compared to the Δ*cshC* mutant, it could suggest that translation efficiency was improved in the double mutant. To further examine this, we took advantage of puromycin, an antibiotic which interacts at the A-site of the ribosome and becomes incorporated in the elongating peptide chain. At sub-MIC, the general translation can be monitored using anti-puromycin antibodies [46–48]. Compared with the WT, a strong decrease in overall translation was observed in the Δ*cshC* mutant (Fig. 3). However, the puromycin incorporation was approximately two-fold higher in the Δ*cshC*, Δ*dinG* double mutant as compared with the Δ*cshC* mutant. It should be noted that the overall translation efficiency was five-fold lower in the Δ*cshC*, Δ*dinG* double mutant compared to the WT and the Δ*dinG* mutant.

Our data suggest that removal of DinG in the Δ*cshC* mutant improves translation efficiency *in vivo* as shown by the increased number of monosomes and polysomes as well as an improved overall translation (Figs 2 and 3, and Supplementary Fig. S3).

### The inefficient processing of the 5′-end of 5S rRNA in the Δ*cshC* mutant can be restored by removal of DinG

**Figure 4.**
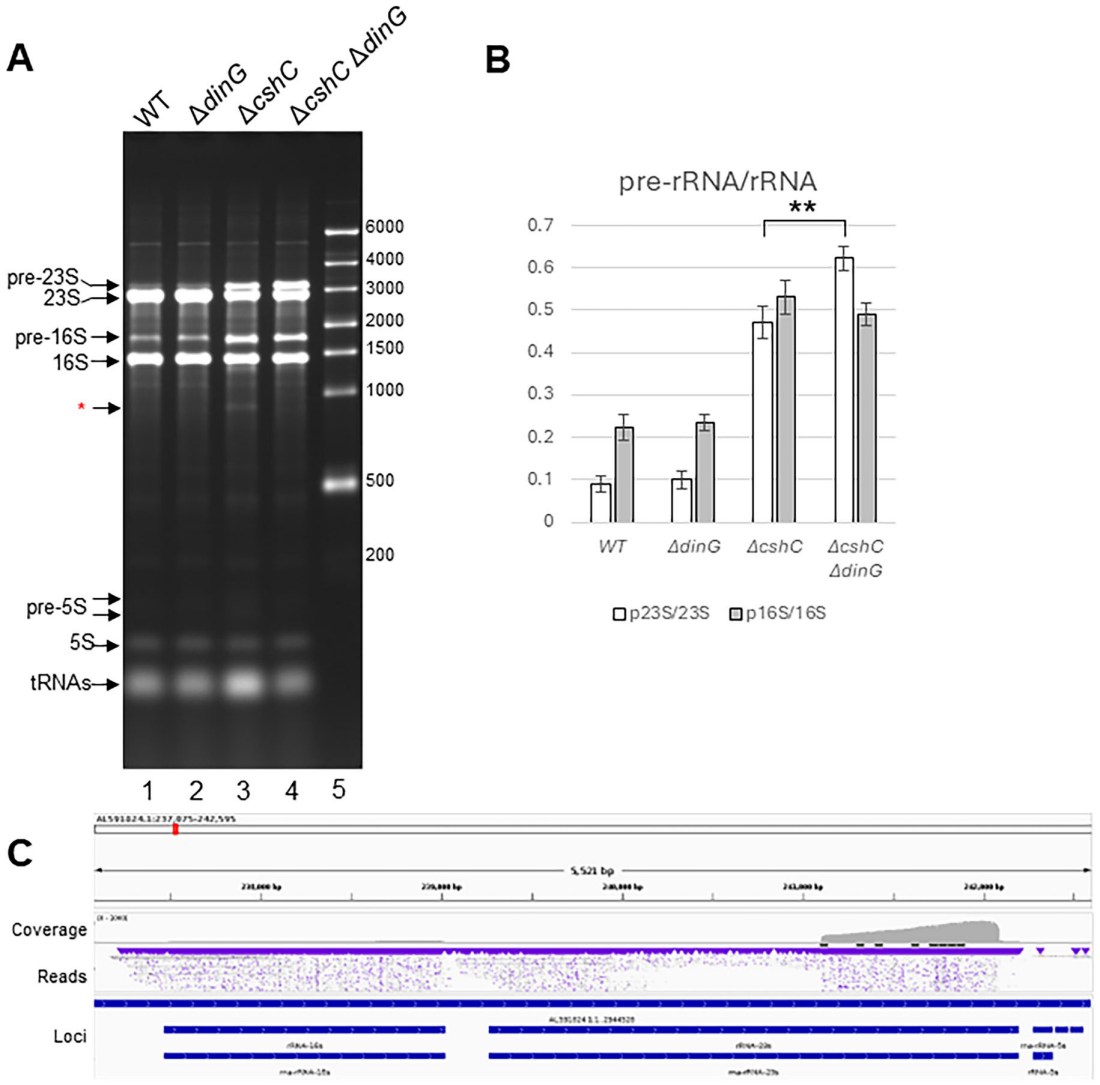
Strains lacking CshC have more unprocessed rRNA species. (**A**) Separation of total RNA from indicated strains (lanes 1–4). Transfer RNAs, pre-23S, 23S, pre-16S, 16S, pre-5S, and 5S rRNAs are indicated with arrows as well as a 23S rRNA-derived 900-nts long RNA-species (red asterisk). Size marker with indicated sizes is shown in lane 5. (**B**) Quantification of pre-rRNAs (p23S and p16S) and rRNAs (23S and 16S) levels from panel (A). Expression is shown as ratio of pre-rRNAs/rRNAs (*n* = 3). The error bars indicate standard deviation, Unpaired two-tailed *t-*test, **P < .005. (**C**) Chromosomal location showing the relative position of the 900-nt long RNA-species in relation to 16S, 23S, and 5S rRNA (see Supplementary Figs S4–S6 for further information).

**Figure 5.**
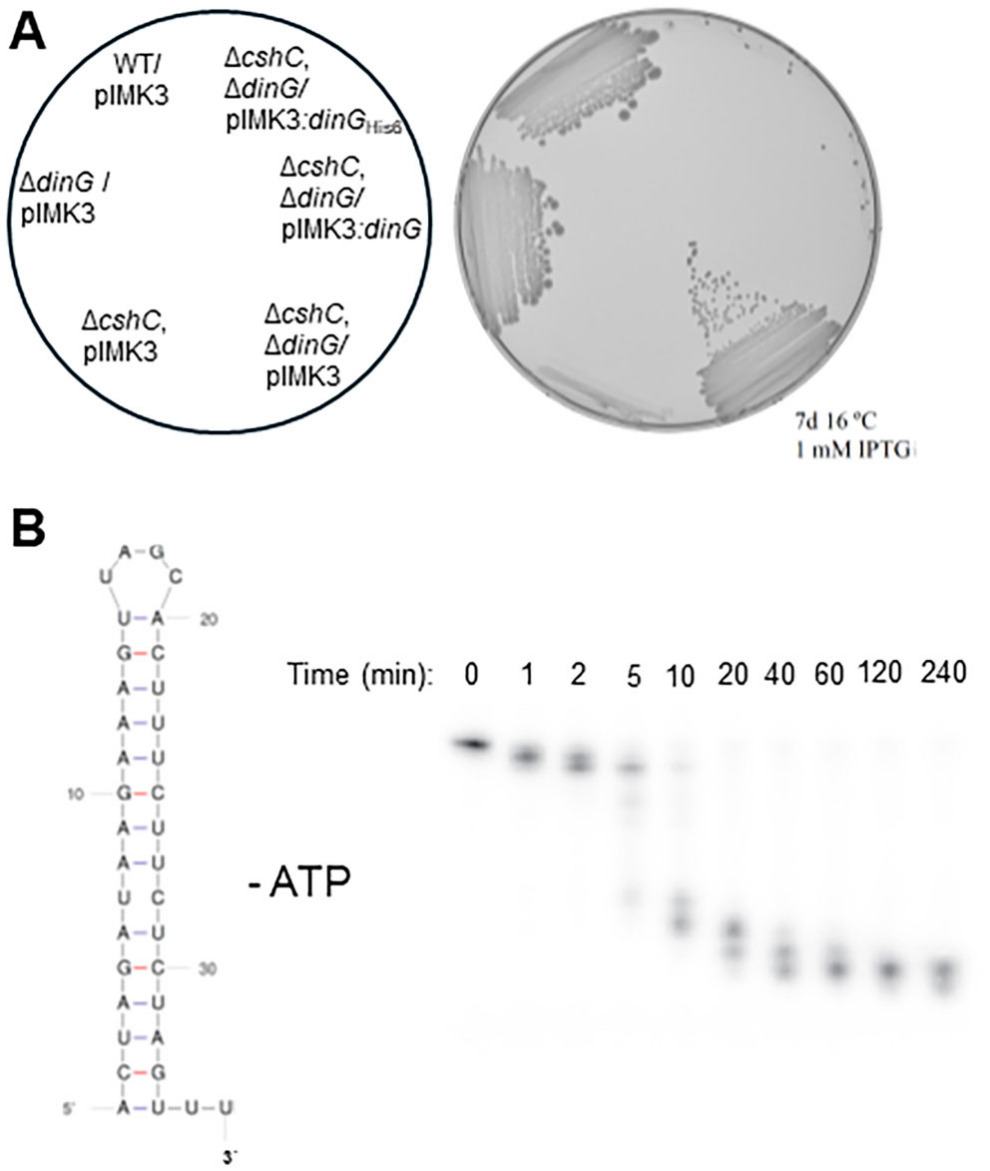
DinG is a 3′- to 5′-exonuclease using both DNA and RNA as a substrate. (**A**) Complementation of the Δ*cshC*, Δ*dinG* double mutant strain by inducible expression of indicated DinG variants_._ Indicated bacteria were streaked on a BHI agar plate supplemented with kanamycin and 1 mM IPTG to induce the *dinG* expression. Plates were incubated at 16°C for 7 days before scoring. (**B**) *In vitro* DinG nuclease activity. The substrate (left image) shows the bottom stalk of the suggested self-complementary 5′ and 3′ ends of the 23S precursor RNA (see Supplementary Fig. S6 for further information). DinG (100 nM) was incubated for indicated time points at 20°C with 10 nM 5′ ^32^P-labeled substrate. Reaction mixtures were separated on 20% denaturing polyacrylamide gels.

To further understand how removal of DinG in a Δ*cshC* mutant could suppress growth defect and increase the number of mature and active ribosomes, total RNA extracts from bacteria grown at 20°C were separated on an agarose gel to compare RNA processing differences between different strains. Of particular interest were RNA-species observed in the Δ*cshC* mutant and the Δ*cshC*, Δ*dinG* double mutant: These strains showed an increased amount of unprocessed 16S and 23S rRNA (Fig. 4A and B). Two additional observations were made: first, an RNA species with a size of ~900 nucleotides was observed in the Δ*cshC* mutant only. By RNA extraction and direct sequencing as well as northern blot, the fragment was identified as part of the 3′-half of the mature 23S rRNA (Fig. 4C and Supplementary Fig. S4). Second, an RNA species connected to unprocessed 5S rRNA was observed exclusively in the Δ*cshC* mutant (Supplementary Fig. S5A). Radiolabeled oligonucleotides targeting the 5′-end and the 3′-end, respectively, of unprocessed 5S rRNA showed that the 5S rRNA was not processed in the Δ*cshC* mutant as compared to the WT and the Δ*dinG* mutant (Fig. 5B). Strikingly, correct processing of the 5′-end of the 5S rRNA was restored in the Δ*cshC*, Δ*dinG* double mutant. Since the 3′-end of the 23S rRNA lies adjacent to the 5′-end of the 5S rRNA on the unprocessed rRNA operon transcript (Supplementary Fig. S6), we next analyzed whether 23S rRNA processing was also affected. The 3′-end of 23S rRNA was improperly processed in the Δ*cshC* mutant, but unlike the case for the 5′-end of 5S rRNA, the processing of the 3′-end of 23S rRNA was not restored in the Δ*cshC*, Δ*dinG* double mutant (Supplementary Fig. S5B, lanes 1–4).

In *B. subtilis*, RNase M5 has been shown to be required for precise processing of the 5S rRNA [49, 50]. We therefore considered the possibilities that a) DinG would interfere with the pre-5S rRNA processing pathway orchestrated by RNase M5, and/or b) unprocessed 5S rRNA would be responsible for the growth defect observed in the Δ*cshC* mutant. To examine the first hypothesis, different mutant combinations were created and grown at 20°C before 5S processing products were monitored by gel electrophoresis and northern blotting. In agreement with the results observed in *B. subtilis*, the absence of RNase M5 in *L. monocytogenes* abolished processing of the 5′-end of the 5S rRNA (Supplementary Fig. S5A and B, lanes 5–8, [49]). Since a similar pattern of unprocessed 5S rRNA was observed in the Δ*cshC* mutant (but not in the Δ*cshC*, Δ*dinG* double mutant) it suggested that DinG in that strain background, either directly or indirectly, inhibited accessibility or activity of RNase M5 to its 5S rRNA target or alternatively, DinG and M5 would work independently on the 5S rRNA target. To examine whether the impaired processing of 5S rRNA was causing reduced growth of strains lacking CshC, relevant strains were plated and incubated at 16°C (Supplementary Fig. S5C). The Δ*rnmV* mutant grew as the WT on agar plates at low temperatures, showing that inefficient processing of the 5′-end of the 5S rRNA did not account for the reduced growth rate observed in the Δ*cshC* mutant (Supplementary Fig. S5C).

### DinG shows 3′ to 5′ exonuclease activity

To further understand the role of DinG and examine its putative exonuclease activity, an IPTG-inducible DinG_His6_ construct was created for purification and *in vitro* analysis. To ensure that the activity of DinG remained despite having six histidines at the C-terminal part of the protein, the Δ*cshC*, Δ*dinG* double mutant was transformed with the DinG_H6_ construct. Expression of the DinG_H6_ and the DinG_WT_ (lacking the histidine tag) constructs in the Δ*cshC*, Δ*dinG* double mutant gave a growth deficiency phenotype as observed in the Δ*cshC* mutant, suggesting that DinG_H6_ and DinG_WT_ could restore DinG functionality in the Δ*cshC*, Δ*dinG* double mutant (Fig. 5A). To examine the importance of the exonuclease activity as well as the role of a putative ATPase/helicase domain, different DinG variants were constructed. Two different DinG variants predicted to lack 3′- to 5′-exonuclease activity were created, where one (DinG_D10A, E12A_), carried the same amino acid substitutions (D10A and E12A) which abolished exonuclease activity of DinG in *S. aureus* [39]. The second variant (DinG_D155Y_) carried the same amino acid substitution in the DEDD-motif as observed in one of the suppressor mutants (Fig. 1A and B). Since a conserved DEAD-box-like element is essential for ATPase/helicase activity, a DinG mutant (DinG_E465A_) carrying a disrupted D_464_E_465_A_466_H_467_-motif was also constructed [51–53]. After purification, the *in vitro* activity of the different DinG variants was assessed using 5′-labeled single-stranded RNA and single-stranded DNA oligonucleotides carrying 10 repetitions of adenine and cytosine (AC)_10_ and (dAdC)_10_, respectively (Supplementary Fig. S7). AC repeats were used as substrate for DinG since the sequence contains repetitions of purines and pyrimidines and the ssRNA/ssDNA are not likely to form any secondary structures, neither by base-pairing to another oligonucleotide nor to itself. No exonuclease activity was observed when examining the activity of the DinGD_10A, E12A_ and the DinG_155Y_ proteins on the ssRNA and the ssDNA oligonucleotides (Supplementary Fig. S7B). In contrast, the DinG_H6_ and DinG_E465A_ proteins showed a prominent 3′-5′ exonuclease activity, strongly indicating that the putative ATP hydrolysis motif located in the helicase-like domain was not required for exonuclease activity. For subsequent experiments, we therefore decided to use the DinG_H6_ and the DinG_D10A, E12A_ proteins (hereafter named DinG and DinG_exo-_, respectively). We next set to examine the substrate specificity of DinG. Although listerial DinG could act as a 3′- to 5′-exonuclease for both DNA and RNA, DNA was the preferred substrate which has been shown previously for other DEDD superfamily exonucleases such as RNase T, but not *S. aureus* DinG (Supplementary Fig. S7C) [39, 54]. However, it has been suggested that the nuclease activity of DinG could be structure- and/or sequence-context dependent. For instance, DinG from *S. aureus* showed a decreased nuclease activity when encountering runs of uracil [39].

Since DinG was required for the generation of the 900 nts long fragment of the 3′-part of the 23S rRNA and since it acts at the site of 23S and 5S processing (Fig. 4 and Supplementary Figs S5 and S6), we were interested to examine whether DinG could act at the pre-23S rRNA “stalk” (Supplementary Fig. S6). In the alpha-fold prediction of the DinG structure (Supplementary Fig. S1), we also included the pre-23S rRNA stalk. The pre-23S rRNA stalk is formed by RNase III, generating a short tail of several unpaired nucleotides at the 3′-end [55]. Strikingly, the 3′-located uracils were predicted to lie closest to the active site as well as amino acids identified through the suppressor mutant screening (Supplementary Fig. S1). Therefore, 23S rRNA stalks, with or without an uracil “toehold” at the 3′-end were synthesized and analyzed (Fig. 5and Supplementary Figs S7 and S8). Our data indicate that DinG indeed shows a 3′ to 5′ exonuclease activity at the pre-23S rRNA stem without a requirement of a toehold (Fig. 5B and Supplementary Fig. S8A). The activity of *S. aureus* DinG has been shown to be reduced in the presence of ATP [39]. We were however unable to observe a similar ATP-mediated repression of *L. monocytogenes* DinG (Supplementary Fig. S8B). Although showing a distinct 3′- to 5′-exonuclease activity, it could be hypothesized that DinG could also exhibit a 5′- to 3′-exonuclease activity and/or endonuclease activity. To assess this, we analyzed the DinG activity on these two modified substrates. First, we labeled the 3′ end of the pre23S_ends RNA oligonucleotide (Supplementary Table S3) by ligating [32P]pCp. This modification both adds the radioactive label and introduces a nonradioactive phosphate to the 3′ end of RNA. Second, we used a 5′ radioactively labeled RNA substrate 23S_ends_5xPTO (Supplementary Table S2) with 5 consecutive phosphorothioate linkages introduced at the 3′ end. Both 3′ terminal phosphate and phosphorothioates have been shown to inhibit 3′-5′ exonucleases [56–60]. No cleavage of the substrate could be observed by DinG using either of these substrates, strongly suggesting that DinG indeed acts exclusively as a 3′- to 5′-exonuclease (Supplementary Fig. S8C).

Considering the putative mechanistic action of DinG at the ribosomal subunits, we were interested to examine whether DinG interacted with the ribosome either in presence or absence of CshC. When examining DinG distribution in sucrose gradients, we were unable to observe a strong interaction between DinG and any of the ribosomal subunits, regardless of whether CshC was present or not (Supplementary Fig. S9). Instead, the majority of DinG was detected in the cytoplasmic fraction. This suggests that DinG binding to ribosomes may be weak or transient.

### DinG selectively targets unprocessed rRNA *ex vivo*

**Figure 6.**
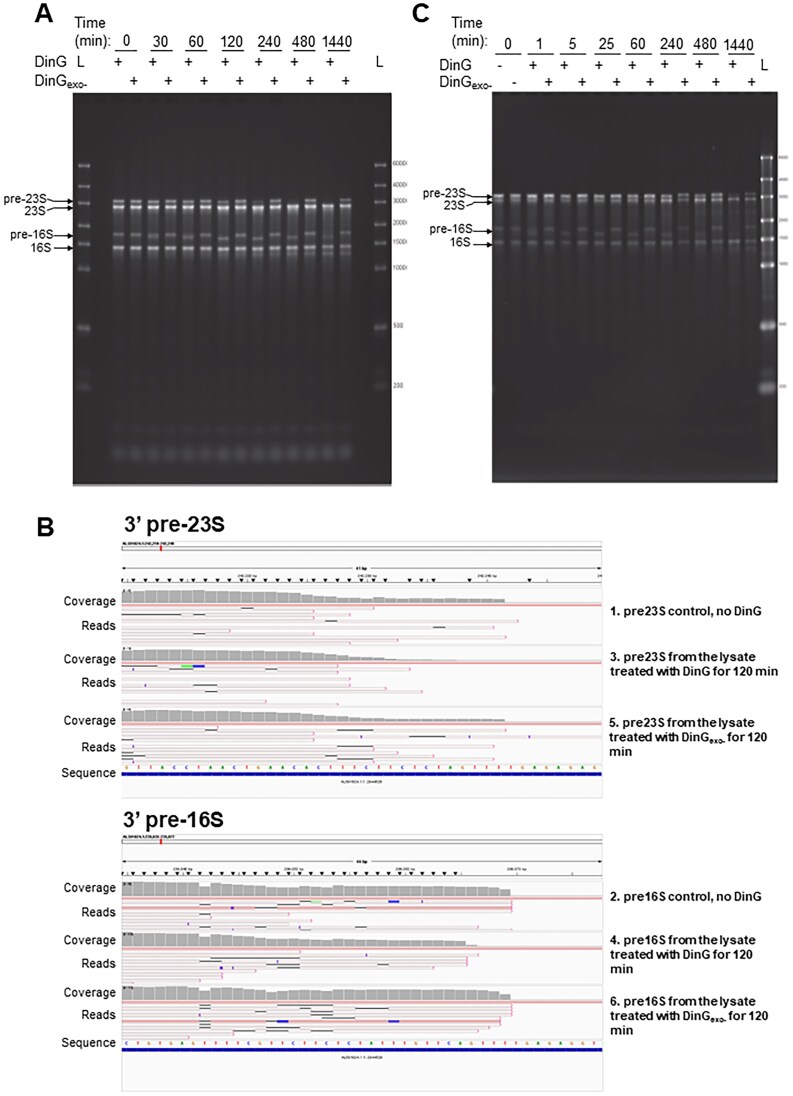
DinG selectively targets unprocessed rRNA in *ex vivo* cell lysates. (**A**) Activity of DinG in whole cell lysates of Δ*cshC*, Δ*dinG* bacteria. The DinG and the DinG_exo-_ proteins were added at a final concentration of 500 nM to lysates from a Δ*cshC*, Δ*dinG* mutant. Reactions were incubated at 20°C for indicated time points up to 1440 min (24 h) before being separated on an agarose gel. L denotes ladder. Fragments 3, 4, 5, and 6 were excised from the gel and sequenced as described in panel (B). The image is a representative of three replicates. (**B**) Base resolution of 3′ part of pre-23S rRNA (upper panel) and pre-16S rRNA (lower panel) after *L. monocytogenes* Δ*cshC*, Δ*dinG* lysate treatment with DinG or DinG_exo-_. Gel-purified RNA fragments were sequenced using Oxford Nanopore direct RNA sequencing methodology to confirm the identity and sequence boundaries of rRNA precursor bands. Sequencing results and alignments were visualized using IGV. The sequence coverage of 3′ ends of 23S and 16S precursors are shown. Sequenced fragments 1 and 2 (marked to the left of both upper and lower panels) were obtained from *L. monocytogenes* Δ*cshC*, Δ*dinG* total RNA and not exposed to DinG. Fragments 3, 4, 5, and 6 were extracted using RNA from Δ*cshC*, Δ*dinG* lysates after 2-h treatment with either purified DinG (fragments 3 and 4) or DinG_exo-_ (fragments 5 and 6) as indicated and described in panel (A). (**C**) DinG exonuclease activity using a ribosomal fraction. The DinG and the DinG_exo-_ proteins were added at a final concentration of 100 nM to a fraction (#13) of the Δ*cshC*, Δ*dinG* lysate separated in 15%–30% sucrose gradient (shown by red asterisk in Supplementary Fig. S3). Reactions were incubated at 20°C and samples were taken at indicated time points for up to 1440 min (24 h), before being separated on an agarose gel.

In light of our observations that rRNA processing is different in the Δ*cshC* mutant compared with the Δ*cshC*, Δ*dinG* double mutant, we next investigated whether purified DinG could affect the levels of processed and unprocessed rRNA in bacterial lysates. To do so, cell extract was prepared from the Δ*cshC*, Δ*dinG* double mutant grown at 20°C before addition of DinG or DinG_exo-_. Separating RNA on non-denaturing gels showed that the mature 16S and 23S rRNA were relatively stable even 24 h after addition of DinG (Fig. 6A). In contrast, within an hour, DinG caused the 16S rRNA precursor to migrate faster, indicative of DinG mediated processing. Surprisingly, sequencing of the 16S rRNA precursor identified only a four-base shortening at the 3′-end of the DinG treated compared with DinG_exo-_ treated sample (Fig. 6B). DinG mediated degradation of the 23S rRNA precursor also involved removal of the 3′-end uracils but no change in gel migration was observed. As expected, DinG_exo-_ did not affect the appearance of either processed or unprocessed rRNA (Fig. 6A).

### DinG acts on unprocessed rRNAs *ex vivo*

A Δ*cshC*, Δ*dinG* double mutant has an increased amount of misfolded ribosomal subunits and unprocessed rRNA (Fig. 2 and Supplementary Fig. S3). To examine whether DinG only acts at aberrantly formed ribosomal subunits, a fraction (#13–@corresponding to pre-50S ribosomal subunits) obtained from the sucrose gradient of the Δ*cshC*, Δ*dinG* double mutant (Supplementary Fig. S3, red asterisk) was examined with and without functional DinG. Surprisingly, the most striking effect was observed at the pre-16S rRNA: presence of DinG but not DinG_exo-_ resulted in processing of the pre-16S rRNA already after 1 min, while leaving the mature 16S rRNA intact for >24 h (Fig. 6C). Unprocessed 23S rRNA identified in the pre-50S ribosomal subunit was also processed, but at a much slower rate compared with the pre-16S rRNA processing.

### DinG selectively targets unprocessed rRNA *in vivo*

**Figure 7.**
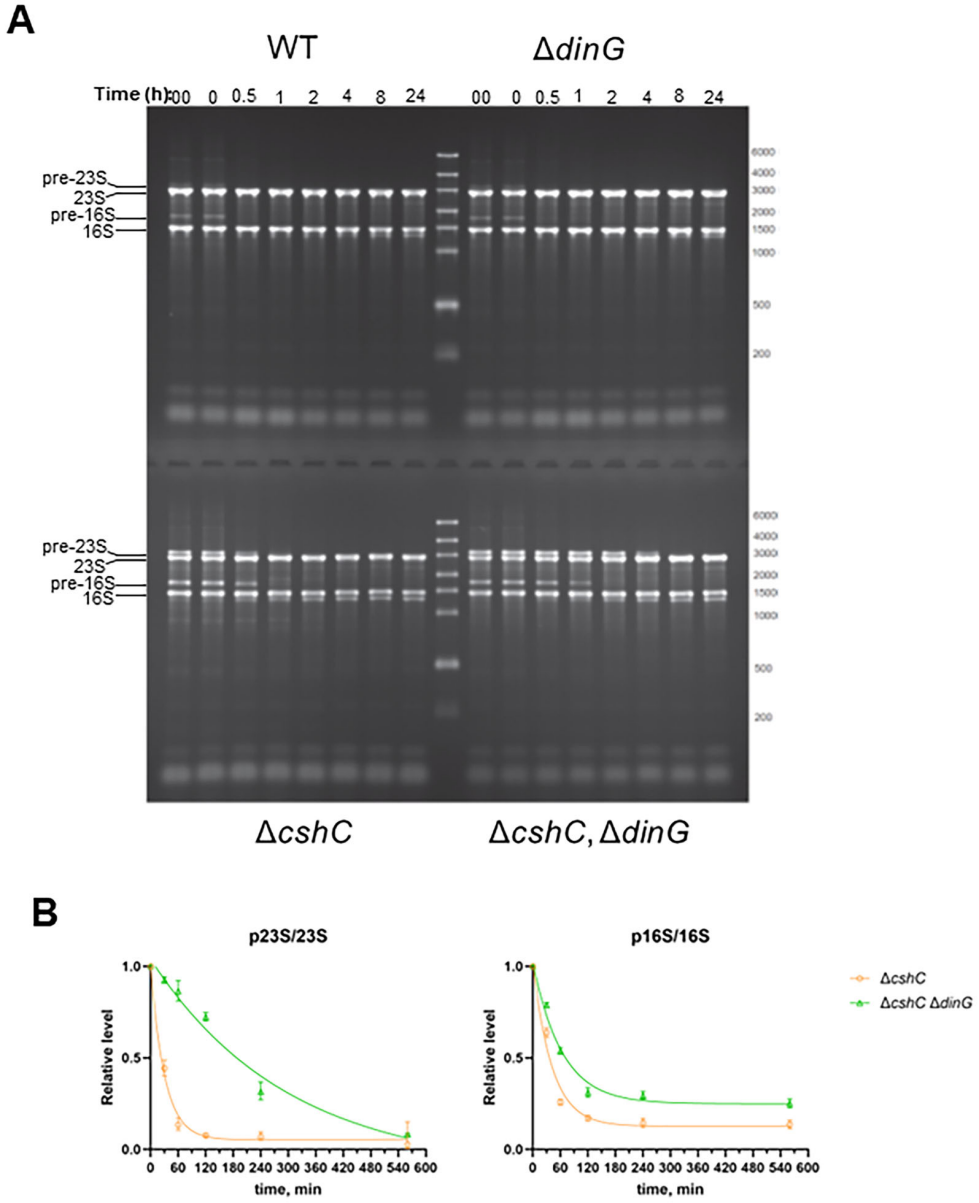
Absence of DinG stabilizes unprocessed rRNA in strains lacking CshC. (**A**) RNA stability in WT; Δ*dinG*; Δ*cshC* and Δ*cshC*, Δ*dinG* strains. Bacterial cultures were grown in BHI at 20°C until mid-logarithmic growth phase when rifampicin was added to stop *de novo* transcription (timepoint 0). Aliquots from each bacterial culture were harvested at indicated time-points for RNA isolation before separation on agarose gels and staining with ethidium bromide. Timepoint marked ‘00’ indicates a sample taken before rifampicin addition (*n* = 3). (**B**) Quantification of relative half-lives of pre-RNAs in the Δ*cshC*, and Δ*cshC*, Δ*dinG* mutants detected in panel (A) (*n* = 3). The error bars indicate standard deviation. The precursor rRNA stability experimental data were fitted with exponential decay curve using GraphPad Prism v10 to estimate the half-lives of these RNA species.

To examine whether DinG affects processing and stability of rRNA also *in vivo*, bacteria were grown at 20°C before addition of rifampicin which inhibits *de novo* transcription. Samples were removed at different time points, total RNA prepared and analyzed on agarose gels. In agreement with the *in vitro* data, mature rRNA was relatively stable in any strain even after 24 h (Fig. 7A and B). The levels of immature precursors of 16S rRNA and 23S rRNA were elevated in the Δ*cshC* mutant and the Δ*cshC*, Δ*dinG* double mutant as compared with the WT and the Δ*dinG* mutant (Fig. 4A). However, both the 16S rRNA precursor and especially the 23S rRNA precursor were more stable in the Δ*cshC*, Δ*dinG* double mutant compared with the Δ*cshC* mutant (Fig. 7A and B). For instance, almost all 23S rRNA precursors were degraded within 2 h in the Δ*cshC* mutant whereas the levels of these precursors were not affected in the Δ*cshC*, Δ*dinG* double mutant at the same time-point. The estimated half-life of pre-23S rRNA was 21 min in the Δ*cshC* mutant whereas it was 207 min in the Δ*cshC*, Δ*dinG* double mutant. For the pre-16S rRNA, the half-life did not vary as much as for the pre-23S rRNA (29 and 44 min, for the Δ*cshC* mutant and the Δ*cshC*, Δ*dinG* double mutant, respectively). Our *in vitro* and *in vivo* stability data thus suggest that DinG selectively targets unprocessed rRNAs but leaves processed rRNA intact.

## Discussion

It has previously been shown that bacteria lacking DEAD-box RNA-helicases display a growth phenotype and a defective formation of mature and active 70S ribosomes [11, 12]. In this paper, we show that removal of the nuclease DinG in a strain lacking the RNA-helicase CshC partially suppresses the growth defect and increases the fraction of mature 70S ribosomes and polysomes.

DinG acts as a dual 3′ to 5′ RNase-@DNase, being able to selectively target unprocessed, immature rRNA whilst leaving processed, mature rRNA (rRNA subunits) intact. In the Gram-negative bacterium *Escherichia coli*, DinG was shown to act as a DNA-helicase, being critical for efficient replication across highly transcribed regions [41]. DNA-helicase activity in DinG from *E. coli* requires a FeS cluster binding domain [61]. In the Gram-positive bacterium *Staphylococcus aureus*, DinG harbors an N-terminal nuclease domain, conserved in Gram-positive bacteria, giving DinG a 3′to 5′ exonuclease activity [39, 44]. Although DinG from *L. monocytogenes* prefers DNA as substrate *in vitro*, it specifically targets unprocessed (but not processed) rRNA *in vivo* and *in vitro* (Figs 6 and 7).

The amount of monosomes and polysomes in the Δ*cshC*, Δ*dinG* mutant was higher compared to the Δ*cshC* mutant (Fig. 2). Since both strains primarily carried processed rRNA of correct size in the monosomes, it could indicate that the rRNA in the 70S ribosomes and polysomes in the Δ*cshC* mutant is accurately processed but that the ribosome (following lack of RNA-helicases) is not assembled correctly, hence giving reduced translation efficiency (as shown in Fig. 3).

The activity of DinG is not sufficient to completely remove all rRNA observed in unprocessed ribosomal subunits (Fig. 6). The complete removal of unprocessed rRNA most likely is mediated by other RNases, possibly requiring endonuclease cleavage (e.g. RNase III, Mini-III) and activity of other exoribonucleases such as RNase R and PNPase which were previously shown or suggested to function in ribosome turnover [9, 10, 55, 62–66]. A product generated through DinG-initiated processing, but likely also activity by other RNases, is represented by the 900 nts fragment of the 23S rRNA observed *in vivo* (Fig. 4A and Supplementary Fig. S4).

Our data implies that DinG and RNase M5 are involved in the processing of the pre-5S rRNA, and that the processing of the pre-5S rRNA by RNase M5 becomes more pronounced in the Δ*cshC*, Δ*dinG* double mutant as compared to the Δ*cshC* mutant (Supplementary Fig. S5). Since DinG does not appear to bind to the ribosome (Supplementary Fig. S9) the above findings indicate that DinG and RNase M5 do not directly interact but instead participate in the same pathway (e.g. inhibition of an enzyme involved in rRNA processing). For instance, DinG could affect binding of the ribosomal protein L18. L18 has been shown to be strictly required for M5 activity in *B. subtilis*, where it potentiates the RNA substrate (the 5S stalk) for M5 recognition [67]. Exactly how DinG inhibits M5 accessibility/activity remains to be determined and whether it also includes L18 and/or the 5S stalk [49, 68].

The lack of other listerial RNA-helicases (e.g. CshB) also causes aberrant ribosomal maturation and reduced growth rate [12]. A Δ*cshB* mutant displays a higher amount of 30S subunit but fewer unprocessed 23S rRNA molecules as compared to the Δ*cshC* mutant. Removal of DinG in the Δ*cshB* mutant increased growth rate but not as much as removal of DinG in the Δ*cshC* mutant (Fig. 1D). This suggests that divergent pathways leading to aberrant ribosomal subunits can initiate DinG activity and removal of unprocessed rRNA.

Our data show that DinG selectively initiates degradation of unprocessed, but not processed, rRNA *ex vivo* and *in vivo* (Figs 6 and 7). *In vitro* (in absence of ribosomal proteins), the lower part of the pre-23S stalk is processed by DinG (Fig. 5B and Supplementary Fig. S8). We therefore suggest that DinG 3′ to 5′ exonuclease activity is halted by ribosomal proteins or complex rRNA structures that reside in mature ribosomal subunits. Maturation of the 23S rRNA in *B. subtilis* requires the double-stranded specific endoribonuclease Mini-RNaseIII, which in turn requires ribosomal protein L3 for correct positioning [69, 70]. It will be of interest to examine whether action of DinG involves Mini-RNaseIII and/or L3. In *E. coli*, the endoribonuclease YbeY has been shown to be required for proper processing of the 16S rRNA but also for degradation of defective 70S ribosomes [63, 71].

In total extract and “pre-50S” extract from the Δ*cshC*, Δ*dinG* double mutant, we observed a large shift in mobility of DinG processed pre-16S rRNA as compared with the unprocessed 16S rRNA (Fig. 6). Surprisingly, sequencing of the fragment showed that only 4 uracil-bases at the 3′-end were removed from the unprocessed 16S rRNA upon DinG treatment. The reason for the large mobility shift is unclear but could indicate structural alterations of the pre-16S rRNA upon DinG processing. The effect of DinG on the pre-23S rRNA is less pronounced, possibly because other RNases important for pre-23S rRNA processing is less active or is lost during ribosome fractionation.

**Figure 8.**
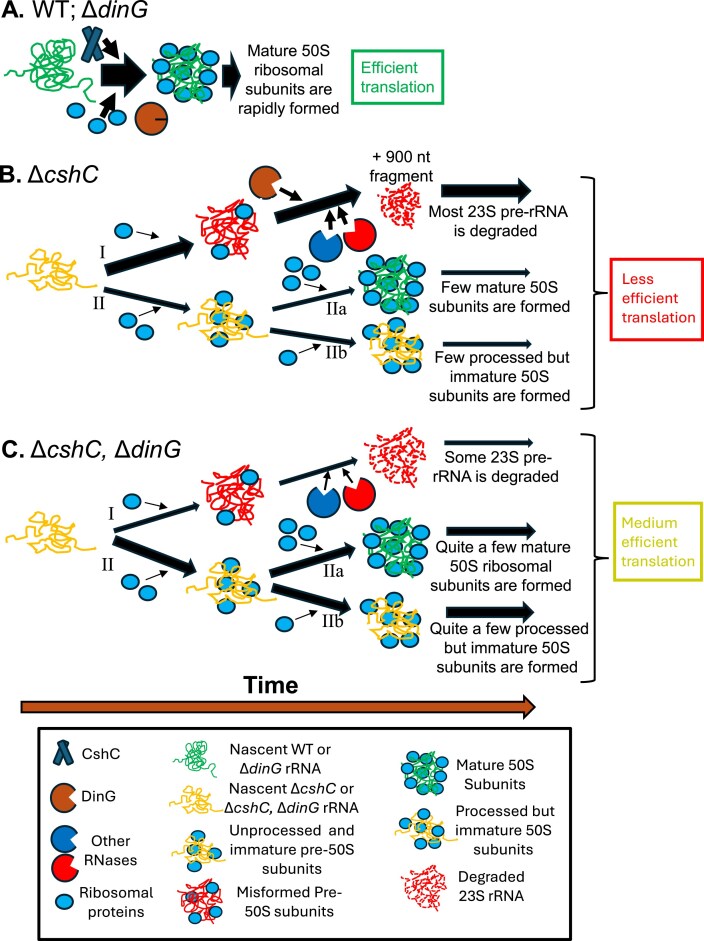
A schematic model of the DinG function. For simplicity, only 23S rRNA and 50S ribosomal subunits are shown. (**A**) Maturation of ribosomal subunits is very fast in the WT preventing exonuclease activity of DinG. Similarly, DinG is redundant in strains carrying intact RNA-helicases. (**B**) In absence of CshC, the ribosomal maturation is delayed. This allows DinG to initiate exonucleolytic activity on pre-rRNA. Other RNases most likely continue processing and degradation of the pre-rRNAs (path I). Ribosomal subunits being able to mature are scarce (path IIa). Alternatively, the ribosomes are processed but not fully mature (due to absence of CshC) reducing their ability to initiate translation (path IIb). (**C**) In a strain lacking both CshC and DinG, most ribosomal subunits will have time to eventually mature (although at a slower rate compared to the WT; path IIa) or alternatively, be processed but still immature (path IIb). Both path IIa and IIb increase translation. Some unprocessed rRNAs will be processed/degraded by other RNases (path I). See text for further details.

We put forward the following working model (Fig. 8). The fate of 50S subunits is shown for simplicity. DinG is a ribosomal quality control exonuclease that initiates degradation of unprocessed rRNA in ribosomal subunits. Upon slow ribosomal maturation (i.e. dysfunctional assembly of ribosomal proteins and RNA structures, which is observed in strains lacking different RNA-helicases, such as CshC), DinG will interact with the misassembled pre-50S subunits to initiate degradation of unprocessed and immature 23S rRNA (path I). After initial processing by DinG, other RNases participate in the degradation of the rRNA. A few rRNAs will have time to follow path II, where they can be fully matured even in absence of CshC (path IIa) or alternatively, be processed but not fully matured (path IIb). For path IIb, the processed but not fully matured ribosomal subunits will still be partially functional, but less efficient than “WT” ribosomal subunits. Translation will be less efficient.

In absence of both CshC and DinG, initial DinG-mediated processing of the rRNA will not occur (path I). Fewer immature and unprocessed rRNAs will be degraded. Instead, more immature rRNA will have time to be processed and slowly mature to functional ribosomal subunits (path II). Some 23S rRNAs could be fully matured (path IIa) or alternatively, be processed but not fully matured (path IIb). Absence of DinG thus aids in getting higher numbers of mature, or partially mature but functional, ribosomes by providing higher levels of precursor subunits that normally would be degraded by DinG. In such a scenario, maturation and formation of functional ribosomes will be slower in the Δ*cshC*, Δ*dinG* double mutant background thereby explaining the decreased translation and growth rate compared to the WT. Importantly, DinG only has the time to be operational if the ribosomal maturation is delayed, such as in an RNA-helicase mutant where immature and unprocessed forms of 30S and 50S subunits are present. The exact mechanism by how the pre-23S rRNA (and pre-16S rRNA) can mature in the Δ*cshC*, Δ*dinG* double mutant remains to be elucidated.

A similar scenario has been observed previously, where removal of RNase R enabled growth and close-to-wild type ribosomal maturation of *B. subtilis* lacking the essential RNase YqfG [9]. In that paper, the authors could show that strains lacking YfqG carried a 3′-extension on the 16S rRNA that made the ribosomes less efficient and becoming targets of RNase R mediated degradation. It could be hypothesized that DinG acts in a similar way as RNase R but at the 23S rRNA.

Our study further highlights the important role RNA-helicases play during the assembly of ribosomal subunits and identifies a novel functionality of the firmicute 3′-5′ exonuclease DinG that targets or marks misassembled ribosomes for degradation.

## Supplementary Material

gkaf1446_Supplemental_File

## Data Availability

Sequencing data has been deposited in BioProject under accession number PRJNA1256696 (https://www.ncbi.nlm.nih.gov/bioproject/PRJNA1256696).
