# Supplementary material for: The DinG exonuclease acts as a primary quality controller to remove unprocessed ribosomal RNAs
Source: Nucleic Acids Res. 2026 Jan 6;54(1):gkaf1446. doi: 10.1093/nar/gkaf1446 (PMC12774634; doi:10.1093/nar/gkaf1446)

Supporting material for:

The DinG exonuclease acts as a primary quality controller to remove unprocessed ribosomal RNAs

Karolis Vaitkevičius and Jörgen Johansson

Department of Molecular Biology, Umeå University, Umeå, Sweden

Laboratory for Molecular Infection Medicine Sweden, Umeå University, Umeå, Sweden

Umeå Centre of Microbial Research, Umeå University, Umeå, Sweden

To whom correspondence should be addressed: jorgen.johansson@umu.se

Content:

Supplementary Tables 1-4

Figure legends for Supplementary Figures S1-S9

Supplementary Figures S1-S9

Supplementary Table 1. Bacterial strains used in this study

Strain	Relevant genotype/phenotype	Reference
Escherichia coli DH5α	Cloning host	(1)
Escherichia coli S17-1	*E. coli* strain used for conjugative plasmid transfer to *L. monocytogenes*	(2)
Listeria monocytogenes EGDe	Wild type *Listeria monocytogenes*	(3)
Listeria monocytogenes Δ*cshC*	EGDe deleted of *cshC* (*lmo1722*)	(4)
Listeria monocytogenes Δ*dinG*	EGDe deleted of *dinG* (*lmo1899*)	This study
Listeria monocytogenes Δ*cshC*Δ*dinG*	EGDe deleted of *cshC* and *dinG*	This study
Listeria monocytogenes Δ*rnmV*	EGDe deleted of Ribonuclease M5 (*lmo0187*)	This study
Listeria monocytogenes Δ*cshC*Δ*rnmV*	EGDe deleted of *cshC* and *rnmV*	This study
Listeria monocytogenes Δ*cshC*Δ*dinG*Δ*rnmV*	EGDe deleted of *cshC*, *dinG* and *rnmV*	This study

Supplementary Table 2. Oligonucleotides used in this study

Oligonucleotide name	Sequence 5'-3'	Restriction enzyme sites/Notes
DdinG-Af_BamHI	AATT GGATCC gatgcccaacaagtagccgttg	BamHI
DdinG-Br_KpnI	AATT GGTACC ctgtttcatctgctccacctc	KpnI
DdinG-Cf_KpnI	AATT GGTACC gaa ggt taa atttttcctgatatgac	KpnI
DdinG-Dr_NcoI	AATT CCATGG gagcttgtcgacaattgcttg	NcoI
dinG-F_BamHI	AATT GGATCC gcagtgaaggaaagaggtgga	BamHI
dinG-R_SalI	AATT GTCGA Cttaagatttgaagtcatatcagg	SalI
dinG-R_His6_SalI	AATT GTCGAC ttaGTGATGGTGATGGTGATGaccttccttaaagaattcactcac	SalI
DinG-F_NdeI	AATT CATatg aaa cag aaa cgc tat ata gtc	NdeI
dinG-R_His6_BamHI	AATT GGATCC ttaGTGATGGTGATGGTGATGaccttccttaaagaattcactcac	BamHI
dinG_exo_D10A_E12A-F	gCtttagCaacaacaggaaatcaagcttcacg	Site directed mutagenesis
dinG_exo_D10A_E12A-R	gacgactatatagcgtttctgtttcat	Site directed mutagenesis
dinG_exo_D155Y-F	Tatgcggaagtgacagcaga	Site directed mutagenesis
dinG_exo_D155Y-R	actatccgcacggtgaggc	Site directed mutagenesis
dinG_DEAH_E465A-F	gatgCggcgcatcattttgcg	Site directed mutagenesis
dinG_DEAH_E465A-R	aattactgcaaaagcatatttgggcaatg	Site directed mutagenesis

Dlmo0187-Af_BamHI	AATT GGATCC gatgaccgtgtgtcacctgc	BamHI
Dlmo0187-Br_KpnI	AATT GGTACC cccgctcataattactacatcct	KpnI
Dlmo0187-Cf_KpnI	AATT GGTACC cag gag gaa gaa aat gag taa agat	KpnI
Dlmo0187-Dr_NcoI	AATT CCATGG CGTCGTTCACTTCAGCTAATGG	NcoI
16S_1134_1169_oligoprobe	CAGTCACTTTAGAGTGCCCAACTAAATGCTGGCAAC	Probe for Northern blot
23S_3Half_oligoprobe2	GGGTAACCTGCATCTTCACAGGTACTATAATTTCACCGAGT	Probe for Northern blot
3'pre-23S_oligoprobe	ctagagaagaaagtgttcagttaggtaacttcg	Probe for Northern blot
5'pre-5S_oligoprobe	caatattgagttgttgaaagattgctctctca	Probe for Northern blot
3'pre-lmor06-oligoprobe	gccctaaataattaggacttttcaaatt	Probe for Northern blot.<br>This sequence motif is present in 2 out of 6 rRNA loci in *L. monocytogenes*
$(AC)_{10}$ RNA	ACACACACACACACACACAC	Nuclease activity substrate
$d(AC)_{10}$ DNA	d(ACACACACACACACACACAC)	Nuclease activity substrate
pre23S_ends (RNA)	ACUAGAUAAGAAAGUUAG<br>CACUUUCUUCUCUAGUUU	Nuclease activity substrate
pre23S_ends_blunt (RNA)	ACUAGAUAAGAAAGUUAG<br>CACUUUCUUCUCUAGU	Nuclease activity substrate
pre23S_ends_5xPTO (RNA)	ACUAGAUAAGAAAGUUAG<br>CACUUUCUUCUCU*A*G*U*U*U<br>(Phosphorothioate links are star marked *)	Nuclease activity substrate

Supplementary Table 3. Plasmids used in this study

Plasmid	Description	Source/Reference
pMAD	*Listeria* allelic replacement vector	(5)
pIMK3	IPTG inducible *Listeria* cloning vector, Km^R	P. Casey (6)
pKVA791	*cshC* in pIMK3	(4)
pET11a	T7 polymerase based protein expression vector	Novagen
pKVA1407	*dinG* in pIMK3	This study
pKVA1557	*dinG* C-terminally tagged with a hexahistidine tag in pIMK3	This study
pKVA1620	*dinG* C-terminally tagged with a hexahistidine tag in pET11a	This study
pKVA1622	*dinG* D10A, E12A derivative C-terminally tagged with a hexahistidine tag in pET11a	This study
pKVA1627	*dinG* D155Y derivative C-terminally tagged with a hexahistidine tag in pET11a	This study
pKVA1634	*dinG* E465A derivative C-terminally tagged with a hexahistidine tag in pET11a	This study

Supplementary Table 4. Sequencing data summary for the suppressor mutants that restore the cold growth of *L. monocytogenes* Δ*cshC* strain.

Chromosome	Position	Ref_Allele Fwd_Strand	Alt_Allele Fwd_Strand	Average Depth	Mutation Type	Codon Subst	AminoAcid Subst	Gene	**Δ*cshC***	**Δ*cshC* *dinG*$_{E570*}$**	**Δ*cshC* *dinG*$_{D155Y}$**	**Δ*cshC* *dinG*$_{L377R}$**	**Δ*cshC* *dinG*$_{E570*}$**	**Δ*cshC* *dinG*$_{T119R}$**
NC_003210	1973255	C	A	74	NONSENSE	Gag/Tag	E570*	dinG					A	
NC_003210	1973479	G	A	72	MISSENSE	tCt/tTt	S495F	dinG		A				
NC_003210	1973833	A	C	77	MISSENSE	cTc/cGc	L377R	dinG				C		
NC_003210	1974500	C	A	68	MISSENSE	Gat/Tat	D155Y	dinG			A			
NC_003210	1974607	G	C	67	MISSENSE	aCg/aGg	T119R	dinG						C

References for the Supporting material

1. Bethesda Research Laboratories. (1986) BRL pUC host: *E. coli* DH5α competent cells. *Focus*, **8**, 9.
2. Simon, R., Priefer, U. and Puhler, A. (1983) A Broad Host Range Mobilization System for In Vivo Genetic Engineering: Transposon Mutagenesis in Gram Negative Bacteria. *Nat Biotech*, **1**, 784-791.
3. Glaser, P., Frangeul, L., Buchrieser, C., Rusniok, C., Amend, A., Baquero, F., Berche, P., Bloecker, H., Brandt, P., Chakraborty, T. *et al.* (2001) Comparative genomics of *Listeria* species. *Science*, **294**, 849-852.
4. Netterling, S., Vaitkevicius, K., Nord, S. and Johansson, J. (2012) A Listeria monocytogenes RNA helicase essential for growth and ribosomal maturation at low temperatures uses its C terminus for appropriate interaction with the ribosome. *J Bacteriol*, **194**, 4377-4385.
5. Arnaud, M., Chastanet, A. and Debarbouille, M. (2004) New vector for efficient allelic replacement in naturally nontransformable, low-GC-content, gram-positive bacteria. *Appl Environ Microbiol*, **70**, 6887-6891.
6. Monk, I.R., Gahan, C.G. and Hill, C. (2008) Tools for functional postgenomic analysis of *Listeria monocytogenes*. *Appl Environ Microbiol*, **74**, 3921-3934.

Figure legends for the Supplementary Figures S1-S9

Figure S1. AlphaFold view of DinG in association with a target (pre-23S rRNA stalk). The DinG protein (Q8Y604) was folded at https://alphafoldserver.com/. A. Suggested structure of full-length DinG protein with color-coded predicted local distance difference test (pLDDT) score. Upper part of protein harbors the evolutionary conserved RNA-helicase domain. The lower left part of the protein contains the 3'-5' exonuclease domain found in Firmicutes. B. Catalytic amino acid residues of the DEDD-motif ($D_{10}E_{12}D_{96}D_{155}$) and residues that were identified as cold-growth defect suppressors of the *L. monocytogenes* Δ*cshA* (T_{119}, $D_{155,}$ L_{377} and S_{495}) are marked. Protein structure is visualised as a ribbon of tan colour, nucleotides are depicted as sticks colored according to nucleobase, magnesium ions are green spheres. C. A magnification of the RNase domain with the DEDD-motif and other relevant residues. Residue T_{119} is relatively close to the nuclease active site. Amino acid S_{495} proximity to RNA may suggest its substitution could interfere with the substrate binding. While L_{377} is not in direct contact and facing away from the RNA but is located in an alpha helix that may be involved in substrate binding.

Figure S2. The increased generation time observed in a Δ*cshC* mutant can be significantly decreased in the Δ*cshC,* Δ*dinG* double mutant. **A.** Indicated bacteria were grown in liquid BHI medium at 20 °C. The growth was followed by determining colony forming units (CFUs) over time. **B.** The obtained generation times were calculated from A. The average generation time for each strain was obtained from quadruplicate (n=4) biological replicas.

Figure S3. Ribosomal profiles and rRNA visualization of the Δ*cshC* mutant (upper panels) and the Δ*cshC,* Δ*dinG* double mutant (lower panels). Bacterial cultures of indicated strains were grown at 20 °C. Bacteria were lysed and supernatants separated by ultracentrifugation in 15-30 % sucrose gradients. Light absorbance at 260 nm wavelength was measured to visualize ribosome species in sucrose gradients (top and bottom panels). Identity of peaks are shown (30S, pre-50S, 50S, 70S, respectively). RNA isolated from indicated fractions were separated on agarose gels (mid panels). Fraction 13 isolated from the Δ*cshC,* Δ*dinG* mutant (indicated by a red asterisk) was used to test DinG activity (Figure 6B).

Figure S4. Characterization of the 900 nucleotides long RNA-species derived from the 3' part of the 23S rRNA. **A.** Suggested 2-dimensional structure of the of *L. monocytogenes* 23S rRNA. The image was produced using the R2DT (https://rnacentral.org/r2dt) online tool (76). The 5'-end of the 900 nts fragment (green arrow), the 3'-end of fragment (red arrow) and the location of the 23S rRNA specific oligonucleotide (black line) used in B are shown. **B.** Northern blot of RNA isolated from indicated strains visualizing the 900 nts long fragment (red asterisk). See also Figure 4.

Figure S5. A strain lacking DinG and CshC show reduced levels of pre-5S. **A.** Separation of total RNA from indicated strains (lanes 1-8). tRNAs, pre-23S, 23S, pre-16S and 16S, pre-5S and 5S ribosomal RNAs are indicated with arrows as well as a 23S rRNA derived 900 nts long RNA-species (red asterisk). Size marker with indicated sizes is shown left of gel. **B.** Northern blot analysis of 3' part of pre-23S (upper panel), 5' part of pre-5S (middle

panel) and 3´part of pre-5S (lower panel) rRNA in indicated strains. **C.** Absence of RNase M5 does not influence bacterial growth. Bacteria were streaked on BHI agar plates and incubated at 16 °C for two weeks before scoring.

Figure S6. A graphical representation of the pre-23S – pre-5S rRNA junction. Upper panel: Overview of locus. Oligonucleotide probes used against 3' precursor end of 23S rRNA (blue arrow) and 5' precursor end of 5S rRNA (purple arrow) in Figure 4B is shown. Red lines indicate processing sites. Bottom panel: More detailed view of rRNA processing sites and putative interaction area of DinG. Light-blue box indicates the bottom stalk of the 23S rRNA precursor that is used as a substrate in Figure 5 and Figure S8. Prediction of the rRNA secondary structure were made by MXFold2 and drawn using VARNA (77,78).

Figure S7. Purification and substrate characterization of DinG. **A.** Coomassie stained SDS-PAGE gel lanes showing relative purity of wild type $DinG_{H6}$, $DinG_{H6}$ D10A, E12A; $DinG_{His6}$ D155Y and $DinG_{His6}$ E465A, respectively, used in this work. **B.** Nuclease activity of DinG derivatives using ssRNA and ssDNA oligonucleotide substrates. 10 nM 5' ^{32}P labelled oligonucleotides $(AC)_{10}$ (upper gel) and $d(AC)_{10}$ (lower gel) were incubated with 500 nM of purified DinG variants for 1 h at 25°C. Reaction mixtures were separated on 20 % denaturing polyacrylamide gels to monitor the hydrolysis of substrates by autoradiography. Reactions separated in lanes 1-5 were incubated without ATP, reactions separated in lanes 6-10 contained 1 mM ATP. **C.** DinG nuclease activity using ssRNA and ssDNA oligonucleotide substrates. 10 nM 5' ^{32}P labelled oligonucleotides $(AC)_{10}$ (upper

gel) and $d(AC)_{10}$ (lower gel) were incubated with indicated concentrations of purified wild type DinG protein for 1 h at 25°C. As controls, 80 nM or 20nM of and $DinG_{D10A, E12A}$ ($DinG_{exo-}$) were incubated with ssRNA or ssDNA substrates, respectively. Reaction mixtures were separated on 20 % denaturing polyacrylamide gels to monitor the hydrolysis of substrates by autoradiography.

Figure S8. DinG nuclease activity using pre-23S rRNA as substrate. The substrate comprises the lower part of the "stalk" of the 23S precursor RNA, forming a hairpin structure (See Figure S6). DinG (100 nM) was incubated for indicated time-points at 20 °C with 10 nM of radioactively labelled substrates. Reaction mixtures were separated in 20 % denaturing polyacrylamide gels. **A.** RNase activity using a 5' [^{32}P]-labelled substrate without a 3' single stranded extension (toe-hold). **B.** RNase activity using a 5' [^{32}P]-labelled substrate with a 3´ single stranded extension in presence of 1 mM ATP. **C.** DinG activity on RNA an substrate labelled with [^{32}P]-pCp at 3' end (middle section of the gel), and an RNA substrate [^{32}P] labelled at 5' end but with 5 consecutive phosphorothioate (PTO) linkages introduced at the 3' end (right section of the gel). The left section of the gel shows a control activity of DinG on 5' [^{32}P] labelled RNA substrate without modifications.

Figure S9. Localisation of histidine tagged DinG in sucrose gradient fractions. Western blot images (upper part) of sucrose gradient fractions from the Δ*dinG* mutant (left panels) and the Δ*cshC,* Δ*dinG* mutant (right panels) that both express DinG from an IPTG-inducible plasmid. Bacteria were grown in BHI liquid medium at 20 °C in presence of kanamycin and 1 mM IPTG until A_{600}=0.5 before polysome profiling, separation and fractionation. Graphs of light absorbance at 260 nm are presented to show the ribosome

separation profile in the sucrose gradients. For example, 30S ribosome subunits are enriched in fractions 6 and 7; 50S ribosome subunits are enriched in fractions 10 and 11; and 70 S ribosomes are enriched in fractions 13 and 14.

Supplementary Figure 1

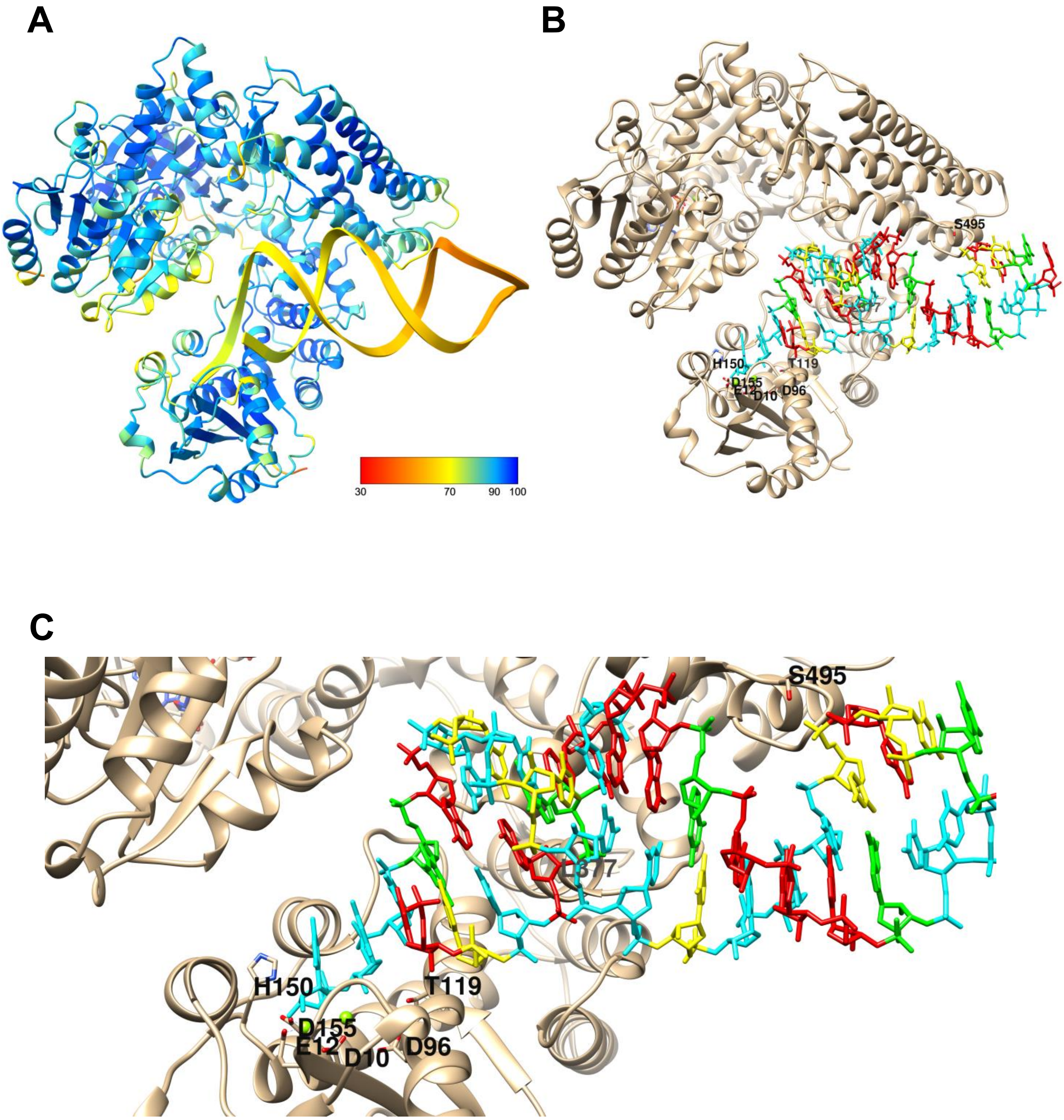

Supplementary Figure 2

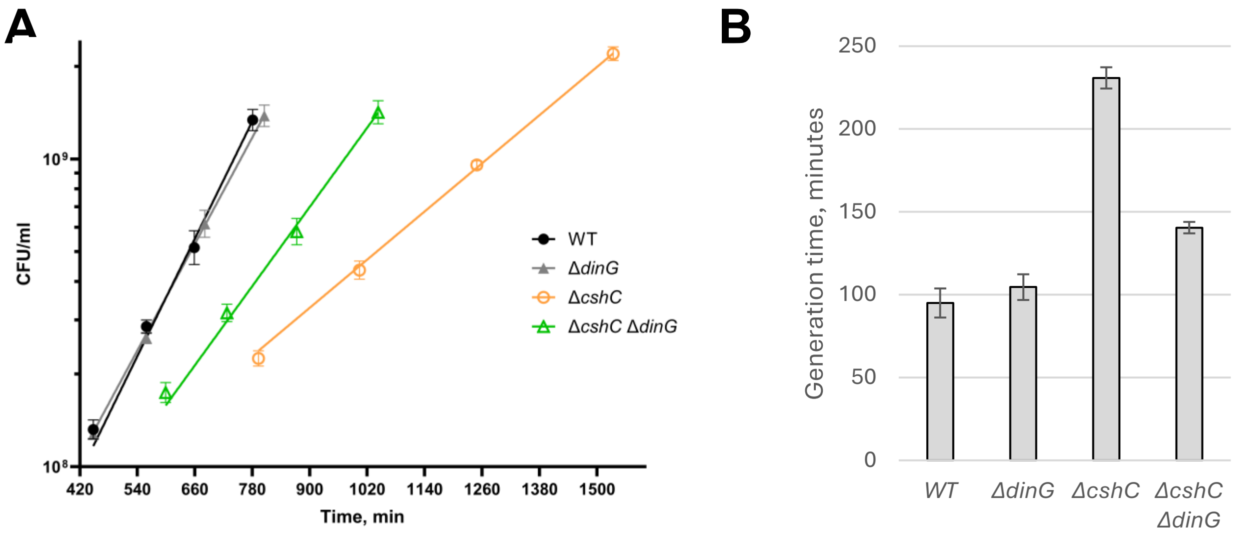

Supplementary Figure 3

Sucrose gradient 15-30%, 22000rpm, 19h

ΔcshC

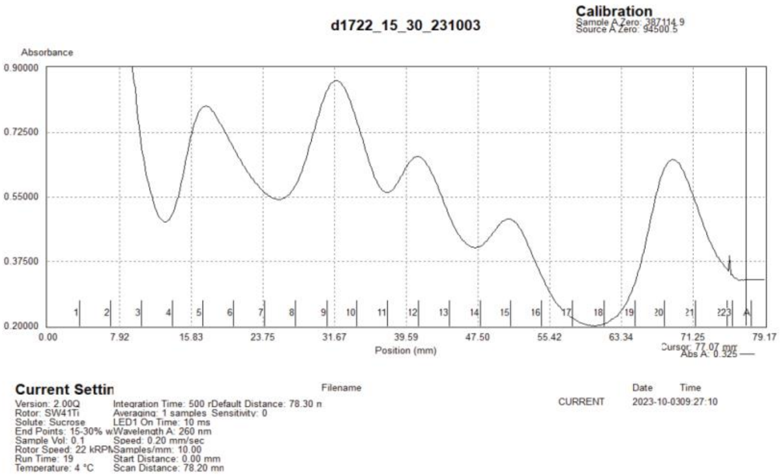

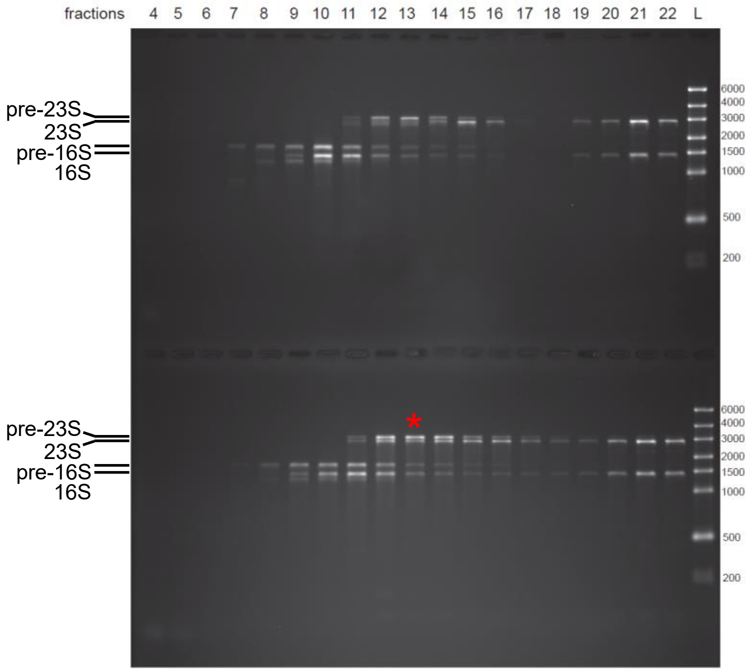

ΔcshC

ΔcshC, ΔdinG

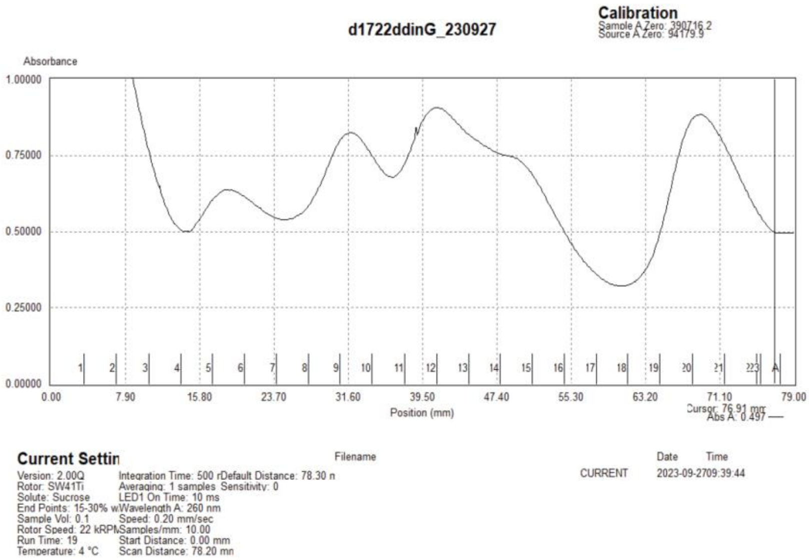

ΔcshC, ΔdinG

Supplementary Figure 4

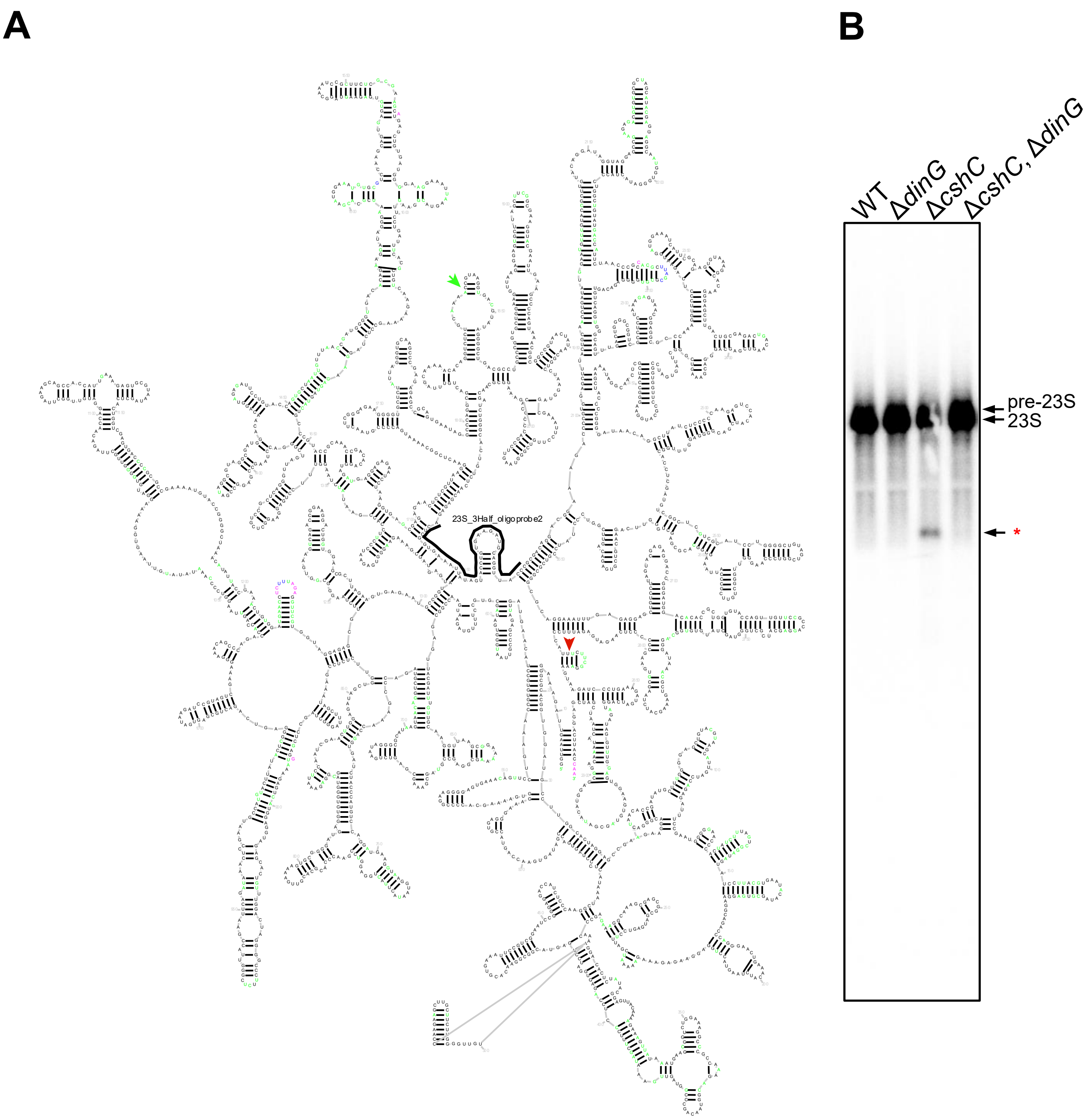

Supplementary Figure 5

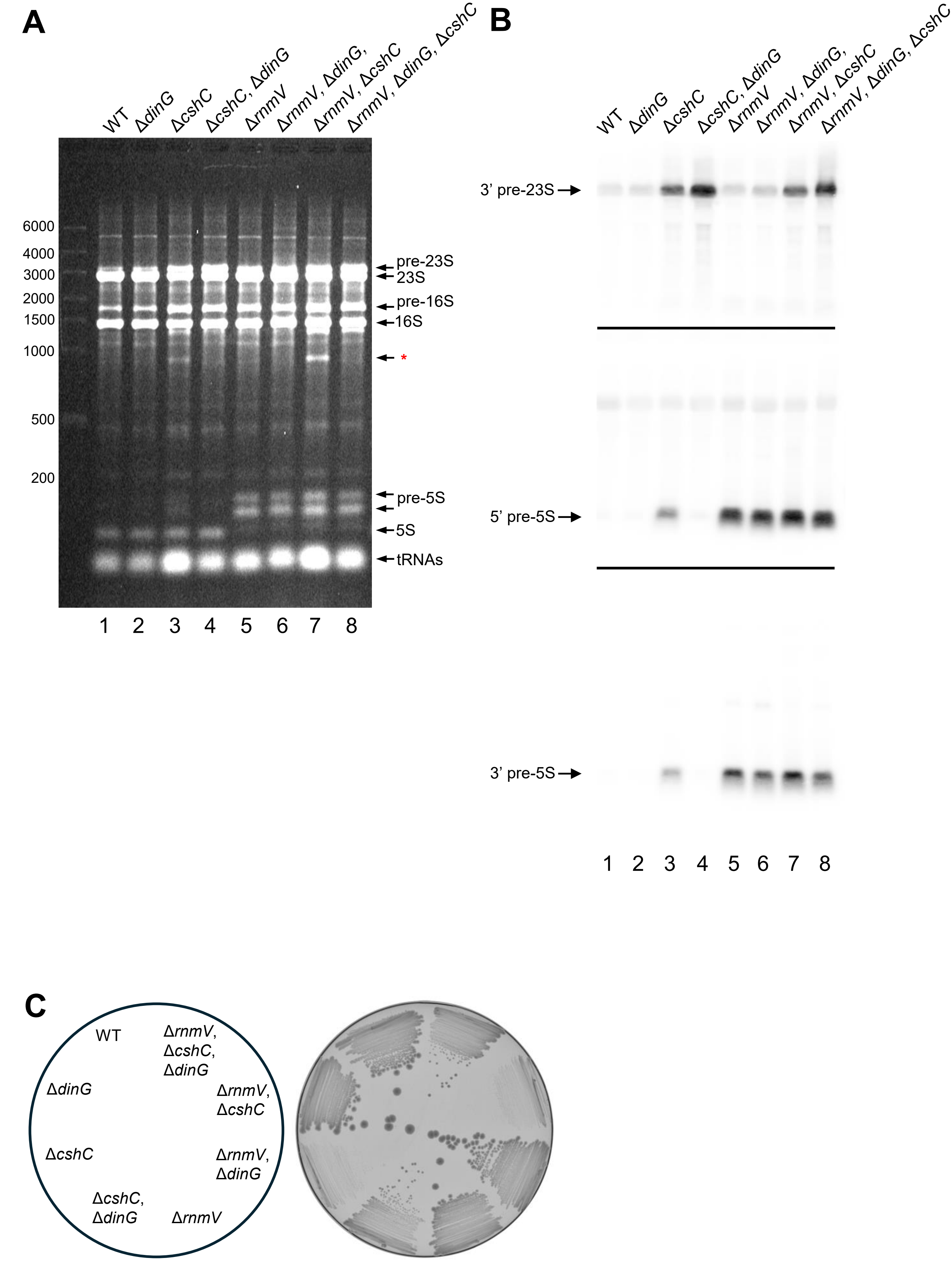

Supplementary Figure 6

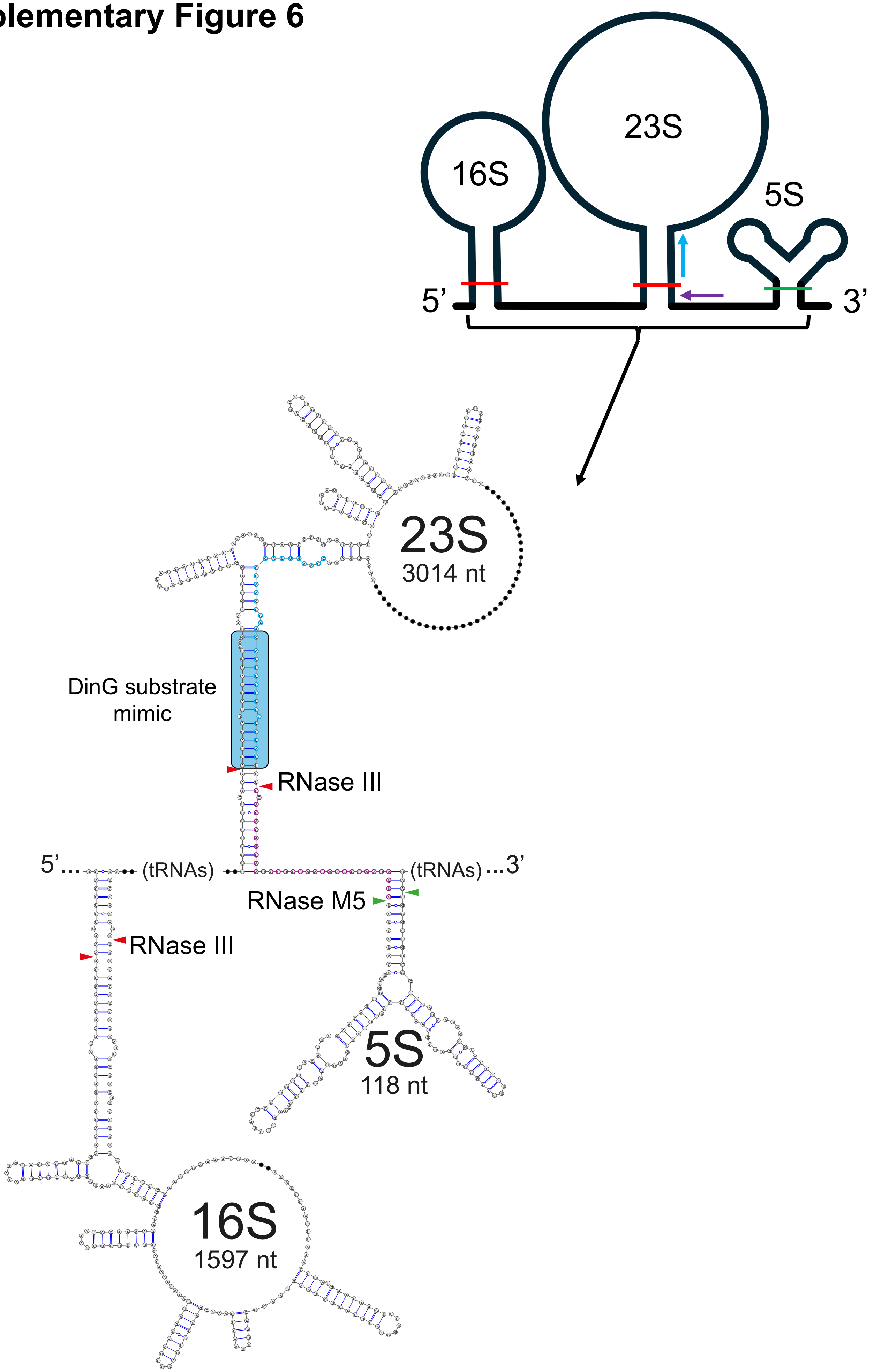

Supplementary Figure 7

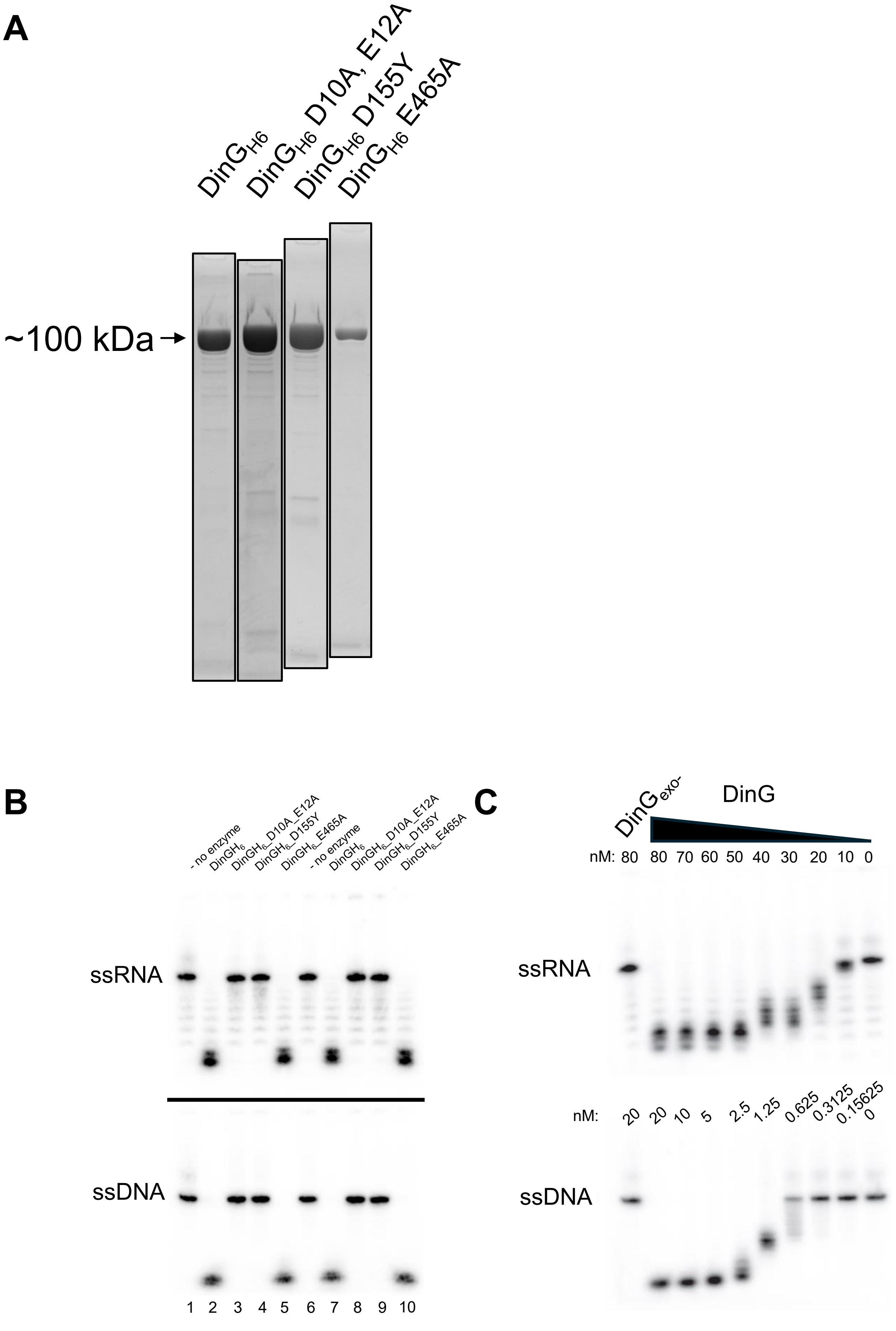

Supplementary Figure 8

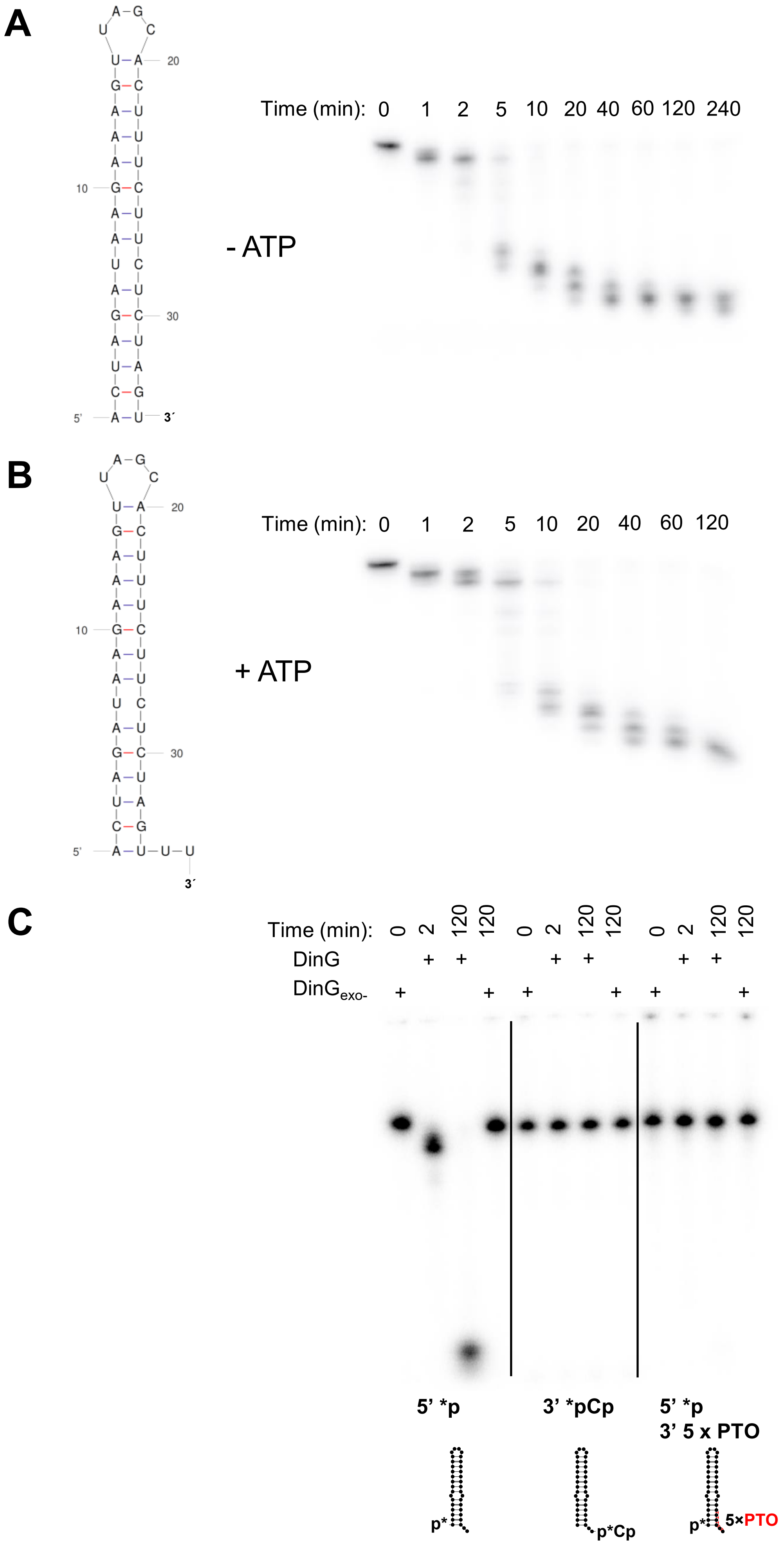

Supplementary Figure 9

Δ*dinG/* pIMK3*:dinG*$_{\mathrm{His6}}$ Δ*cshC*, Δ*dinG/* pIMK3*:dinG*$_{\mathrm{His6}}$

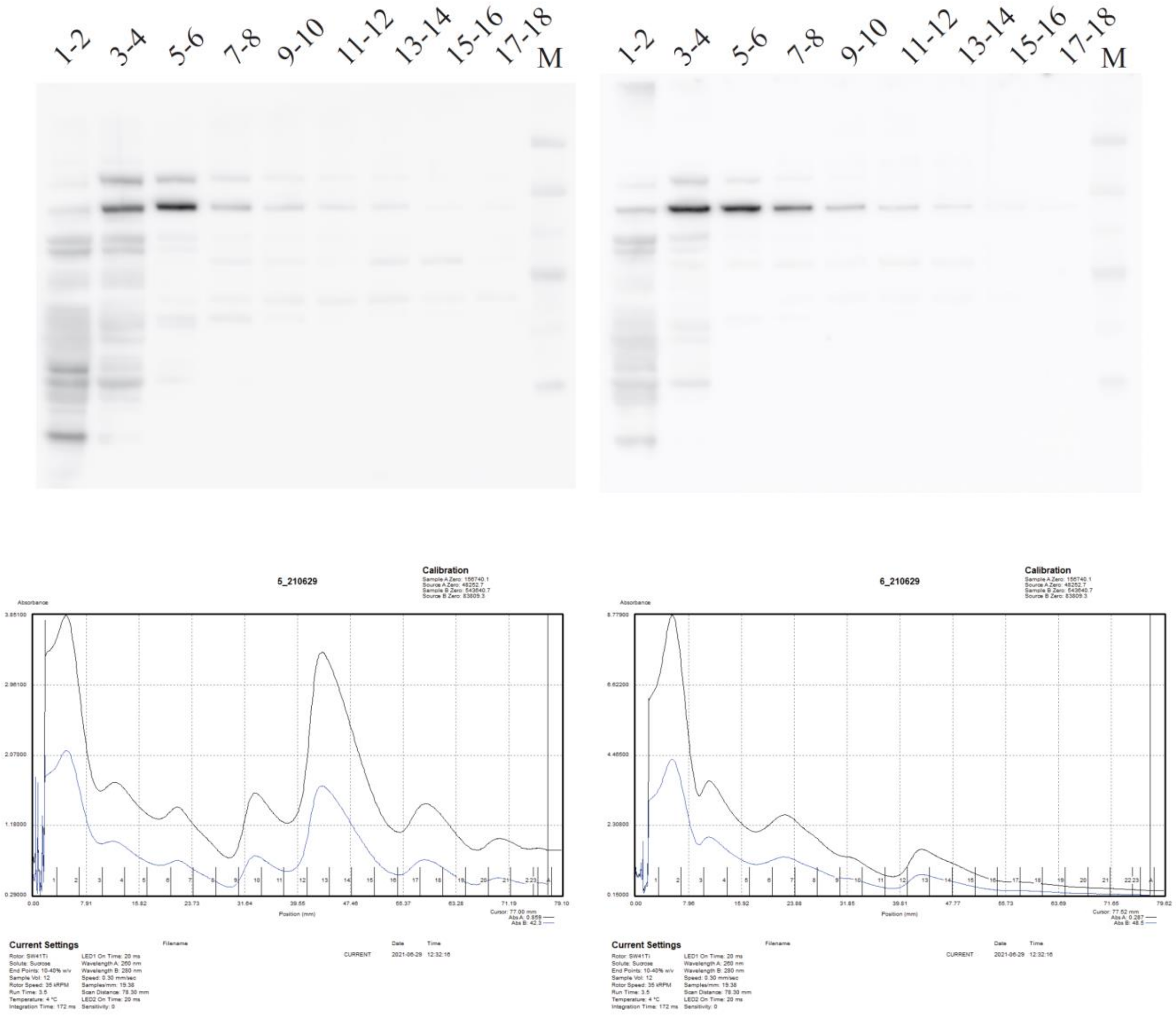

Supplement: gkaf1446_Supplemental_File [file gkaf1446_supplemental_file.pdf]
